# Apolipoprotein E Binding Drives Structural and Compositional Rearrangement of mRNA-Containing Lipid Nanoparticles

**DOI:** 10.1021/acsnano.0c10064

**Published:** 2021-03-23

**Authors:** Federica Sebastiani, Marianna Yanez Arteta, Michael Lerche, Lionel Porcar, Christian Lang, Ryan A. Bragg, Charles S. Elmore, Venkata R. Krishnamurthy, Robert A. Russell, Tamim Darwish, Harald Pichler, Sarah Waldie, Martine Moulin, Michael Haertlein, V. Trevor Forsyth, Lennart Lindfors, Marité Cárdenas

**Affiliations:** † Biofilms - Research Center for Biointerfaces and Department of Biomedical Science, Faculty of Health and Society, Malmö University, 20506 Malmö, Sweden; ‡ Advanced Drug Delivery, Pharmaceutical Sciences, R&D, AstraZeneca, 431 83 Gothenburg Sweden; § Large Scale Structures, Institut Laue Langevin, Grenoble F-38042, France; ∥ Forschungszentrum Jülich GmbH, Jülich Centre for Neutron Science JCNS, Outstation at Heinz Maier-Leibnitz Zentrum, Lichtenbergstraße 1, 85748 Garching, Germany; ⊥ Early Chemical Development, Pharmaceutical Sciences, R&D, AstraZeneca, SK 10 4TG Cambridge, U.K.; # Early Chemical Development, Pharmaceutical Sciences, R&D, AstraZeneca, 431 83 Gothenburg, Sweden; ¶ Advanced Drug Delivery, Pharmaceutical Sciences, R&D, AstraZeneca, CB2 0AA Boston, Massachusetts 02451, United States; □ National Deuteration Facility (NDF), Australian Nuclear Science and Technology Organisation (ANSTO), Lucas Heights, 2232 Sydney, NSW, Australia; ■ Austrian Centre of Industrial Biotechnology, Petersgasse 14, 8010, Graz, Austria; ○ Institute of Molecular Biotechnology, Graz University of Technology, NAWI Graz, BioTechMed Graz, Petersgasse 14, 8010, Graz, Austria; ● Life Sciences Group, Institut Laue Langevin, Grenoble F-38042, France; △ Partnership for Structural Biology (PSB), Grenoble F-38042, France; ▲ Faculty of Natural Sciences, Keele University, Staffordshire, ST5 5BG, U.K.

**Keywords:** lipid nanoparticles, mRNA delivery, ApoE, protein corona, small-angle scattering

## Abstract

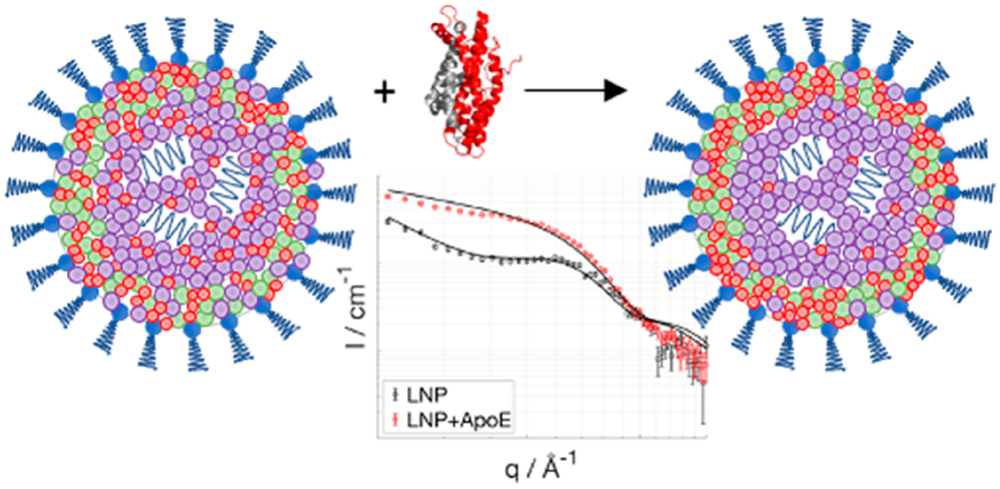

Emerging therapeutic treatments based on the production of proteins by delivering mRNA have become increasingly important in recent times. While lipid nanoparticles (LNPs) are approved vehicles for small interfering RNA delivery, there are still challenges to use this formulation for mRNA delivery. LNPs are typically a mixture of a cationic lipid, distearoylphosphatidylcholine (DSPC), cholesterol, and a PEG-lipid. The structural characterization of mRNA-containing LNPs (mRNA-LNPs) is crucial for a full understanding of the way in which they function, but this information alone is not enough to predict their fate upon entering the bloodstream. The biodistribution and cellular uptake of LNPs are affected by their surface composition as well as by the extracellular proteins present at the site of LNP administration, *e.g*., apolipoproteinE (ApoE). ApoE, being responsible for fat transport in the body, plays a key role in the LNP’s plasma circulation time. In this work, we use small-angle neutron scattering, together with selective lipid, cholesterol, and solvent deuteration, to elucidate the structure of the LNP and the distribution of the lipid components in the absence and the presence of ApoE. While DSPC and cholesterol are found to be enriched at the surface of the LNPs in buffer, binding of ApoE induces a redistribution of the lipids at the shell and the core, which also impacts the LNP internal structure, causing release of mRNA. The rearrangement of LNP components upon ApoE incubation is discussed in terms of potential relevance to LNP endosomal escape.

The development of RNA-based therapies using lipid nanoparticles (LNPs) as delivery vehicles is emerging as a versatile approach with clinical potential. Many companies have understood their significant value and have focused their core development in an LNP-based platform, *e.g*., Acuitas Therapeutics. In 2018, the U.S. Food and Drug Administration (FDA) approved the drug Onpattro based on a small interference RNA (siRNA) targeting transthyretin.^1^ Moreover, Moderna and Pfizer/BioNTech have received emergency authorization in several markets for mRNA-LNPs-based vaccine against SARS-CoV-2.^2−4^

LNPs have been developed as gene vectors to deliver, for example, siRNA, to knock down the production of a specific protein in the body, or mRNA, to produce a deficient protein *in situ*. Despite the great advances in LNP therapies, there are still challenges to translate this type of formulation from siRNA to mRNA, following their differences in size (20 *vs* 1000 nucleotides, respectively) and configuration (double *vs* single stranded, respectively). LNPs can potentially pack more copies of siRNA per nanoparticle compared to mRNA, making them more efficient for this type of therapy.

Upon intravenous administration, apolipoprotein E (ApoE) in blood serum binds to LNPs,^5,6^ which leads to LNP accumulation in the liver.^7,8^ ApoE is a reversible apolipoprotein partially responsible for lipid trafficking in the body: when lipid bound, ApoE binds to LDL receptors in the liver and fats can be recycled. Parallel to the above-mentioned cellular uptake route, LNPs can be internalized *via* other pathways, such as clathrin-mediated endocytosis and macropinocytosis.^9^ The cationic ionizable lipid (CIL) is a critical component in LNPs that quickly concentrates in the liver upon intravenous LNP administration.^7,8^ Microscopy observations have shown the presence of intact LNPs inside the endosomal compartment,^9,10^ in addition to observations that protein expression *in vivo*, upon mRNA-LNP administration, is localized in the liver.^8,11^ Together, these findings are interpreted in terms of the intact LNPs entering the liver cells, which then release mRNA in the cytosol, where the protein expression occurs.^8,11^ Nevertheless, CIL accumulation as a consequence of binding to and extraction by ApoE cannot be excluded, since protein production takes place but remains at a very low level. Maugeri *etal*.^12^ detected a 1:1 CIL to nucleotide ratio in endocytosed extracellular vesicles, while most LNPs are prepared with an excess of CIL, as in this study (3:1 CIL to nucleotide ratio). It remains unknown whether ApoE binding to LNPs plays a role in the endosomal escape and successful delivery of mRNA to the cytoplasm. However, it is clear that ApoE binding to LNPs is critical for cellular uptake and protein production in the liver.^6,13^

CILs, when formulated in LNPs, possess a headgroup with an apparent p*K*_a_ between 6 and 7,^14^ which makes LNPs neutral at extracellular pH (∼7.4), but charged at the lower pH values found in endosomes (6.5–4.5).^15^ This property enables CIL to encapsulate the anionic RNA at low pH; it has been proposed that it also facilitates endosomal escape by fusing with the negatively charged endosomal membrane.^16^ In LNPs, helper lipids, such as cholesterol, phospholipids (*e.g*. distearoylphosphatidylcholine, DSPC), and poly(ethylene glycol) (PEG) lipid, are required not only to stabilize the nanoparticle but also for its function.^11,17,18^ The PEGylated lipid is employed to stabilize the particle and to control its size; smaller LNPs are generated at higher PEG–lipid ratios.^19^ It is suggested that the core–shell structure describes the LNP nanostructure with CIL-RNA being located in the core, the rest of the lipid components being colocalized in the shell.^20−22^ In reality, the lipid component distribution (core *versus* shell) is indeed quite asymmetric, as demonstrated by small-angle neutron scattering (SANS),^20^ with DSPC being mainly segregated toward the LNP shell. Interestingly, the LNP size and surface composition play a major role in the protein expression efficacy.^20^ The distribution of lipids other than DSPC in the LNP, and their potential impact on LNP function, is not yet clear even though the LNP shell is speculated to be cholesterol-rich.^20,23^

The state of the art regarding the role of components in LNP function can be summarized as follows:^8^ (1) cholesterol and the saturated phospholipid DSPC stabilize the LNP surface, as in cellular membranes, (2) CILs help the LNP to bind and fuse with the endosomal membrane, facilitating endosomal escape, (3) PEG-lipids ensure that LNP aggregation does not occur prior to administration. Upon intravenous administration, the PEG-lipid is shed, enabling LNPs to fuse with the endosomal membrane releasing mRNA. This process can be facilitated by using a shorter acyl chain lipid such as dimystoryl phosphatidyl ethanolamine (DMPE) as demonstrated *in vivo*.^11^

In this work, LNPs prepared with deuterated CIL and match-out deuterated cholesterol allowed us to use SANS to determine not only the exact cholesterol, DSPC, and CIL composition and distribution across the LNP shell and core at pH 7.4 but also any potential effect that ApoE binding might have on the various lipid component distribution and the overall LNP structure. The latter is of greater importance since ApoE adsorption to LNPs boosts their cellular uptake in the liver, but may potentially affect LNP endosomal escape and mRNA release upon inducing a change in the surface structure, *i*.*e*., redistribution of the lipid components. SANS in combination with selective deuteration was the technique of choice to study the structure and composition of LNPs. On one hand, SANS gives information on the size and shape of the object in solution (details can be found in the following references^24,25^). On the other hand, the isotopic substitution in the lipids and/or cholesterol enables determining the location of the deuterated component in the particle, together with the tuning of D_2_O content in the buffer. The ability to distinguish between different isotopes derives from the distinct scattering lengths, which, weighted by the molecular volume, are known as scattering length density (SLD). The mixture of H_2_O (SLD 0.56 × 10^–6^ Å^–2^) and D_2_O (SLD 6.35 × 10^–6^ Å^–2^) can be adapted to match out the signal from a selected component; for example, deuterated cholesterol with SLD equal to SLD_D2O_ is called match-out cholesterol.^26^

## Results and Discussion

### Determination of Structure and Composition in the Core and Shell of mRNA- LNPs

**Figure 1:**
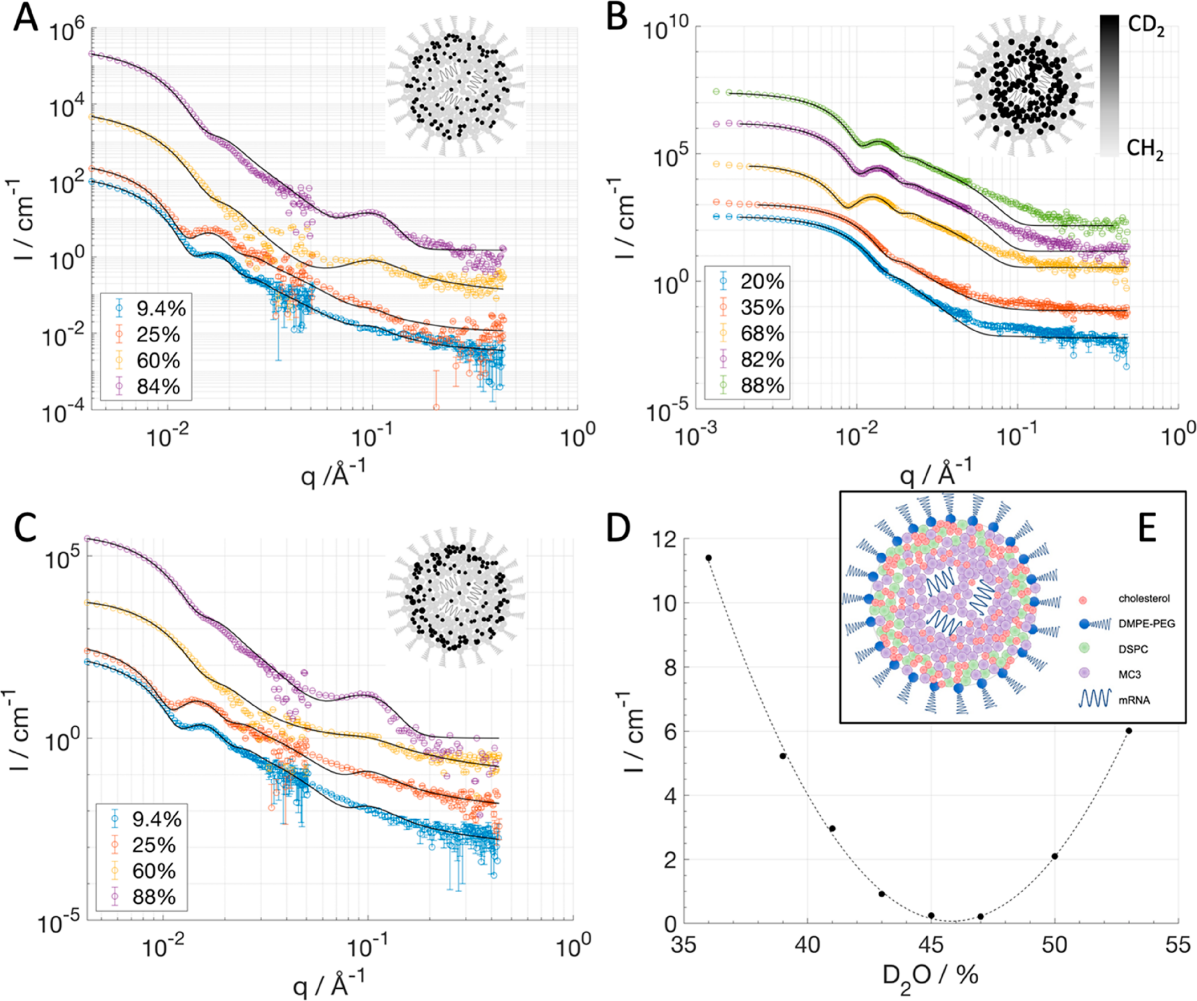
SANS data collected with sample MCH at four different contrasts (A), MMC at five different contrasts (B), and MCHPC at four different contrasts (C). The legends in panels A, B, and C describe the percentages of d-PBS present in the solvent. The black solid lines are the result of model fitting. The curves were shifted for clarity. The exact composition of the LNP formulations is given in Table 1. The peak due to internal structure is clearly visible for MCH and MCHPC (d-PBS > 60%). For MMC, however, a small deviation from the model around *q* = 0.1 Å^–1^ for all solvent contrasts is possibly due to internal structure, which was not included for this particular data set modeling. In panel D, the scattering intensity averaged over the *q* values 0.004–0.007 Å^–1^ is plotted against the percentage of D_2_O content in the buffer for the MMO sample, showing that this sample is invisible in solvents where proteins are also invisible to neutrons. Schematic drawing of the core–shell structure including the distribution of the components in the LNP; water is not represented in the schematics; the core has an average water volume fraction of 18 ± 5% (E). In the insets of panels A, B, and C the LNP schematics have the components colored according to their SLD values (*i.e*., deuterated components are black).

mRNA-containing LNPs were formulated with dilinoleylmethyl-4-dimethylaminobutyrate (DLin-MC3-DMA, usually abbreviated as MC3, the CIL used here), DSPC, cholesterol, mRNA, and DMPE-PEG2000 at the molar ratios given in Table 1. Four different samples were prepared using the same mixing ratios but with various substitution of some components with their deuterated version (Table 1). The percentage of deuterated components in each sample was chosen to highlight the position of a given component with respect to the rest; in particular MCH and MMC had the natural cholesterol and MC3, respectively, fully substituted with the deuterated version. The MCHPC and MMO compositions, in terms of deuterated components, were designed to have the core and shell of the LNP matched, *i*.*e*., the same SLD for the core and shell, so that the LNP turns invisible in a given H_2_O/D_2_O mixture (here, we chose the SLD that matches proteins, *i*.*e*., ∼43% D_2_O). However, the scattering intensity for MCHPC could only be minimized but not matched out in any H_2_O/D_2_O mixture (Figure SI1), while MMO was made invisible in 46% D_2_O (Figure 1D). The structure of the mRNA-containing LNPs was determined by SANS (Figure 1). By using different degrees of deuterium substitution, various parts of the LNP particle are highlighted (shell *versus* core) among the samples. This enables the determination of not only the overall structure but also of the distribution of the different components within the LNP shell and core.

**Table 1:** Composition of LNP Was 10% mol DSPC, 50% mol CIL, 38.5% mol Cholesterol, 1.5% mol DMPE-PEG, 0.015% mol mRNA; Four Samples (Named MCH, MCHPC, MMO, MMC) Were Prepared with Different Levels of Deuteration in mol % in Phosphate Buffer Saline pH 7.4

	% deuterated component			
	MCH	MCHPC	MMO	MMC
cholesterol	100	100	42^a^	0
DSPC	0	32	37	0
MC3 (CIL)	0	0	42	100

a Here d-cholesterol (average 87% D) was used instead of d-cholesterol (average 89% D).

The SANS data in Figure 1A–C were analyzed using either a simultaneous fit for the core–shell sphere model^27^ only (sample MMC) or adding a broad peak model^28^ to better describe the data in the *q* range above 0.05 Å^–1^ (samples MCH and MCHPC). A detailed description of the data analysis can be found in the Supporting Information (SI). Such a broad peak arises from the internal structure of the CIL/RNA packing and mainly from the contrast between the solvent and the lipid components. For MMC at a D_2_O content above 35 vol %, the contrast between the deuterated MC3 and the solvent is quite low (match point is 82 vol % D_2_O), while for lower D_2_O vol % the incoherent background from hydrogen atoms masks the contribution of the internal structure. Thus, no clear broad peak at *q* > 0.05 Å^–1^ is present for MMC. Sample MMO was fully contrast-matched between shell and core and was therefore fitted by a sphere model (see next section and the SI, Figure SI2).

These SANS results confirm the core–shell structure previously suggested for LNPs^20−22^ and provide detailed quantification of the distribution of all four lipid components in the core and shell separately. In all cases, the core radius, shell thickness, and shell scattering length density (SLD_shell_) were the fitting variables with the constraint to be the same among the different solvent contrasts for each sample preparation, while the core SLD (SLD_core_) was allowed to vary accounting for solvent in the core following the relationship SLD_core_ = vf_sol_ × SLD_sol_ + (1 – vf_sol_) × SLD_dry core_. vf_sol_ and SLD_dry core_ are fitting variables and constrained to be unique for each LNP preparation; vf_sol_ is the solvent volume fraction in the core, and SLD_dry core_ corresponds to a weighted average of all LNP components’ scattering length density except solvent. Further details on the fitting procedure and compositional derivations are included in the Methods section and the Supporting Information. In general, consistent structural (Table 2) and compositional (Table 3) information was found for MMC, MCHPC, and MCH. The distribution of the various LNP components across shell and core is depicted schematically in Figure 1E.

**Table 2:** Structural Information on mRNA-LNPs As Determined by SANS^a^

	MCH	MCHPC	MMO	MMC	average and SD
core radius/nm	26.4 ± 0.1	26.7 ± 0.1	b	31.0 ± 0.9	28 ± 3
shell thickness/nm	5.0 ± 0.1	6.9 ± 0.1	b	5.1 ± 0.3	6 ± 1
total radius/nm	31.4 ± 0.2	33.6 ± 0.2	31.5 ± 0.1	36 ± 1	33 ± 2
core solvent fraction (v/v%)	23 ± 1	17 ± 1	b	13 ± 3	18 ± 5
core volume/nm^3^	77 070	79 730	b	124 800	
shell volume/nm^3^	52 610	79 160	b	70 640	
total volume/nm^3^	129 680	158 890	123 600	195 430	
hydrodynamic radius (DLS)/nm^c^	43 ± 1	44 ± 1	41 ± 1	43 ± 1	

a LNPs were formulated according to the molar composition stated in Table 1.

b These parameters cannot be assessed in MMO due to lack of contrast between shell and core in the experimental conditions used.

c Intensity averaged.

**Table 3:** Compositional Information on mRNA-LNPs As Determined by SANS^a^

	shell volume fractions				core “dry” volume fractions		
sample	DSPC^b^	Chol	MC3	DMPE-PEG^b^	Chol	MC3	mRNA^b^
MCH	25.4	32.6 ± 0.2	39.0 ± 0.8	3.0	12.4 ± 0.2	75.2 ± 0.4	12.4
MCHPC	21.9	30.9 ± 0.2	44.6 ± 0.8	2.5	11.1 ± 0.2	74.5 ± 0.4	14.4
MMC^c^	30	36 ± 4	30.5 ± 1.8	3.5	12.2 ± 0.5	76.8 ± 0.2	11.0
average and SD	26 ± 4	33 ± 3	38 ± 7	3.0 ± 0.5	12 ± 1	75 ± 1	13 ± 2

a Volume fractions estimated from the fitted SLD using the core–shell model. Excellent agreement with the experimental results was obtained, and the initial mixing ratios are within experimental error (see Methods section and the SI, Tables SI4 and SI5 for molar fractions).

b The distribution of these components was kept fixed: DSPC and DMPE-PEG in the shell and mRNA in the core.

c The largest error for the composition in MMC relates to higher error in the SLD of the shell (Table SI2).

There is good agreement between the total size measured by SANS and dynamic light scattering (DLS) with a slightly larger radius for MCHPC and MMC (see Table SI1). DLS radii (hydrodynamic radii) are systematically larger than the total radius determined by SANS since the latter is the sphere radius, which is expected to be smaller than its hydrodynamic radius: the hydrodynamic radius reflects the collective motion of particles with its counterion cloud. For MMC, the sample containing 100% deuterated CIL, there is a larger discrepancy in shell and core volume together with the solvent volume fraction in the core. Deuteration can give rise to changes in the lamella repeating distance^29^ besides the known changes in packing of phospholipids^30^ and diffusion.^31^ This is due to changes in the vibrational modes, dipolar moments, and hydrogen bonding upon deuteration. The internal structure of LNPs depends largely on the ionic conditions of the MC3 lipid, and the differences observed in LNP structure may arise from deuteration. Interestingly, despite these differences in total LNP size, only minor deuteration effects in composition are observed (Table 3).

The LNP surface was modeled as a single shell that should contain both the phospholipids and the lipid portion of the PEGylated lipids. This assumption holds valid since the contribution of the highly hydrated PEG layer (hydration ∼61% in mushroom conformation^32^) to the overall shell scattering is negligible. In order to rule out the need of an additional shell, a preliminary analysis of the SANS curves was performed, and the resulting pair distance distribution functions and density profiles supported the choice of a single-shell core sphere model (see Figure SI3 in the SI). The analysis shows that the shell thickness is similar for all samples except for the one containing both deuterated cholesterol and DSPC (MCHPC). This is the sample where the contrast between core and shell is larger compared to other samples, which probably gives a higher neutron sensitivity to this layer (see details on fitted SLD values in Table SI2 in the SI). Interestingly, the shell thickness is larger than a DSPC monolayer (2.7 nm)^33^ and closer to a DSPC bilayer in the gel phase (5.8 nm).^34^ This suggests that a disordered bilayer probably forms due to the presence of high-curvature MC3 (see schematics of the LNP in Figure 1E).

Finally, from the broad peak position in samples MCH and MCHPC, an internal *d*-spacing of 6.35 ± 0.02 nm is found. This distance probably represents the characteristic *d*-spacing of the inverse worm-like micellar structure of the core.^20,28^ This is consistent with previous results obtained on bulk phase samples.^20^

As already described, the various SANS data sets are sensitive to the distribution of each component within the LNP. For example, greater accuracy of our estimation for cholesterol is found in the MCH data set since all cholesterol molecules are deuterated in this case, and they can be distinguished from the rest of the LNP components. MCHPC is less sensitive to the cholesterol distribution since some DSPC is also deuterated but highlights the total shell conformation, knowing that DSPC mostly occupies the shell.^20^ MMC, on the other hand, has greater accuracy toward MC3 distribution since it is the only component being deuterated. In general, the compositional information obtained from the combined SANS data for MCH, MCHPC, and MMC are in excellent agreement (Table 3) and show that the shell volume consists on average of 33 ± 3% cholesterol, 38 ± 7% MC3, 26 ± 4% DSPC and 3.0 ± 0.5% DMPE-PEG. The dry core volume (*i*.*e*., excluding the solvent) is dominated by the MC3, which occupies on average 75 ± 1%, 12 ± 1% cholesterol, and 13 ± 2% mRNA. The high consistency in composition among the samples studied shows that small variations in overall particle size and volume ratio between shell and core do not have a major effect on the component distribution among shell and core in LNPs.

In terms of molar concentration (Table SI4), the cholesterol is approximately 2 times more concentrated in the shell than in the core, while the MC3 concentration is approximately 3 times more concentrated in the core than in the shell. The cholesterol molar ratio in the shell is about 51%, which translates to a ∼2.7 to 1 cholesterol:DSPC molar ratio. This molar ratio is above the solubility limit for cholesterol in PC lipids.^35−37^ However, since the shell also contains CIL and DMPE-PEG, the actual ratio of cholesterol to total lipids is ∼1.1– 1, and the risk for cholesterol crystal formation on the LNP surface should be low (assuming a homogeneous distribution between CIL and DSPC). On the other hand, the core contains approximately 24 mol % cholesterol, which lies just at the measured solubility limit of cholesterol in MC3^20^ and could suggest that no cholesterol crystals would form in the core either. However, for the deuterated cholesterol containing samples, MCH and MCHPC, the estimated SLDs based on volume constraints are lower by 23% and 12% than the best fit SLD values for both core and shell, respectively. This may imply that the SLD value for deuterated cholesterol is slightly higher than the expected value found by mass spectroscopy (MS).^38^ Such a discrepancy could be explained by the formation of two-dimensional cholesterol crystals,^39^ which would decrease the cholesterol molecular volume^40,41^ and hence increase the cholesterol SLD. If this is true, two-dimensional cholesterol crystals could form mainly on the LNP surface as a consequence of, for example, an increased affinity between cholesterol and DSPC compared to MC3, which could potentially lead to phase separation or domain formation.

### Producing a Shell–Core and ApoE Contrast Matched mRNA-LNP

In order to validate the composition determined by independent SANS experiments on samples with different deuteration schemes (Figure 1A–C), an LNP sample was formulated by mixing the deuterated and hydrogenated forms of the three main lipid components in appropriate ratios (Table 1), giving SLD_shell_ = SLD_core_. In parallel, the SLD for the contrast matching point was chosen so that it would also match the ApoE SLD (sample MMO). This condition was selected so that changes in structure and/or composition could be followed upon ApoE incubation. The SLD of ApoE was calculated from the amino acids sequence; accounting for the H/D exchange of labile hydrogens, the protein was found to be matched at 43 vol % D_2_O based buffer. The MMO sample was diluted in solvents with D_2_O content ranging from 36% to 53%, in order to find the optimal matching condition; experimentally this was found to be 46% d-PBS and very close to the match value for ApoE (Figure 1D). The data for the match out study were collected at a single configuration (detector distance and collimation) and over a limited *q*-range. As expected, the simultaneous fit of four selected contrasts is consistent with a sphere where no core–shell structure is visible having an SLD of (2.58 ± 0.15) × 10^–6^ Å^–2^ (data and fit in Figure SI2). From this sample, detailed information on the components’ volume fractions was not accessible due to the shell–core matched out conditions and the limited range of solvent contrasts. However, the ability to completely match out the scattering contribution validates the component distribution found for this particular LNP formulation and the high reproducibility in its composition across sample batches.

In addition, the volume fractions obtained from MMC were used to estimate the SLD of MMO, accounting for the deuteration scheme (Table 1), and the calculated SLD agreed extremely well with the best fit value for the SLD obtained with the sphere model for MMO. In the conditions of MMO, the scattering data were not sensitive to inhomogeneities across the shell and the core, and even in the event of domain formation within the shell, no major intensity would arise since the average SLDs for each component are close. Furthermore, the absence of scattering rules out any segregation effect of deuterated components from hydrogenated ones that would otherwise lead to an intensity increase.

### Binding Isotherm for ApoE to mRNA-LNPs

**Figure 2:**
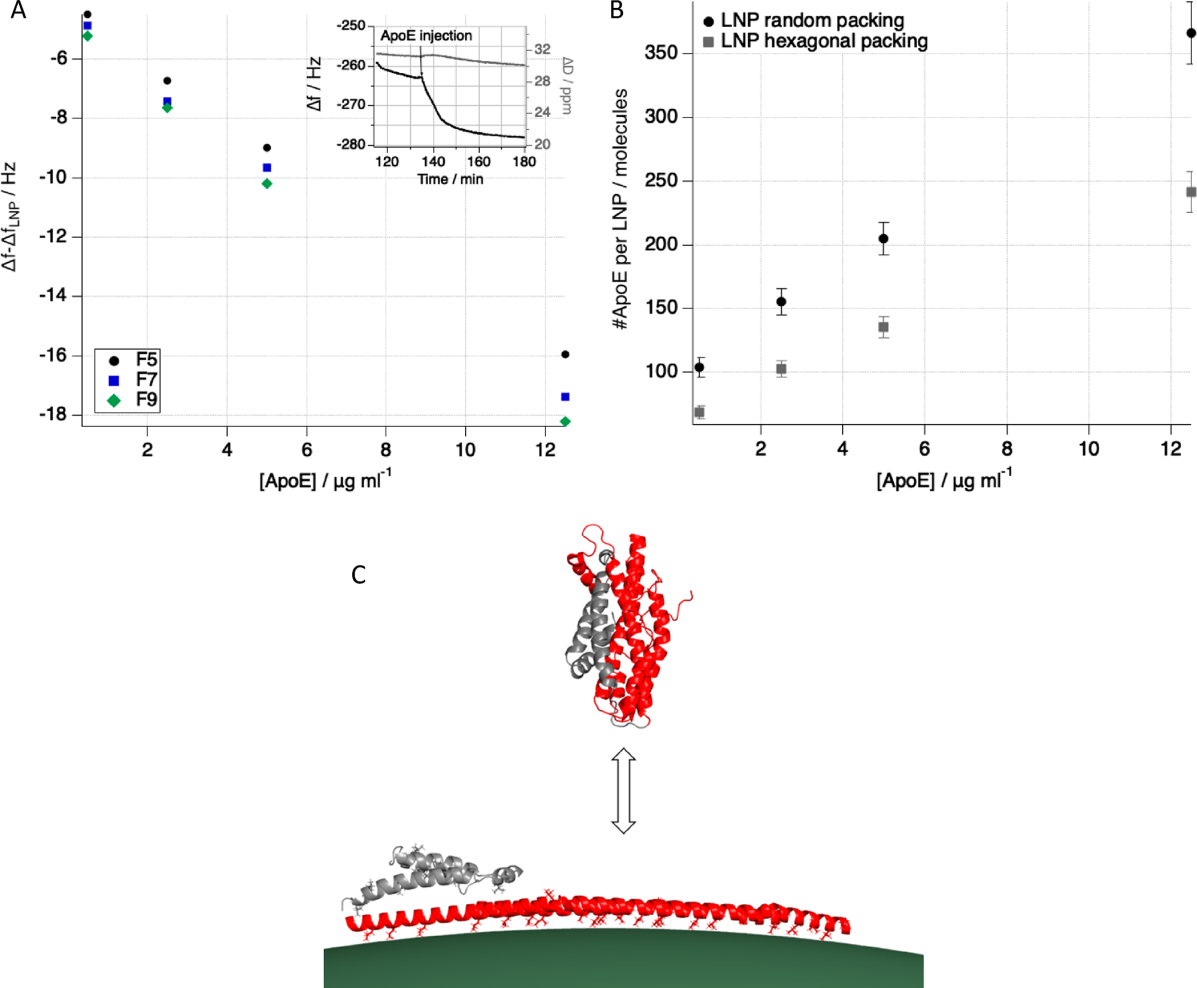
ApoE binding to LNP as measured by LNP immobilized particles on a QCM-D sensor. The raw frequency shift for overtone 5, 7, and 9 is reported as a function of ApoE concentration (A). The frequency shift has been offset by the equilibrium value obtained after LNP injection/rinsing. The overlap for all overtones suggests that the ApoE is a rigid film adsorbed on the LNP (no change in dissipation occurs). Note that a negative change in frequency is related to an increase in adsorbed wet mass. The number of ApoE molecules per LNP as a function of ApoE concentration is calculated assuming hexagonal (gray squares) and random (black circles) packing by using the Sauerbrey equation (B). Transition of free ApoE^44^ (PDB ID: 2l7b) into proposed hairpin-like configuration^43^ adapted to fit on an 80 nm diameter LNP (green). Domains bound to the LNP surface are shown in red with hydrophobic leucines and isoleucines shown as sticks (C). The illustration was prepared using the PyMOL Molecular Graphics System, version 2.0, Schrödinger, LLC.

Prior to determining the structural effect of ApoE binding to LNPs by SANS, the binding isotherm for ApoE to LNPs had to be established. Attempts were made using indirect chromatographic and ELISA type of methods, but the presence of free ApoE complicated the interpretation of the results. Therefore, a quartz crystal microbalance with dissipation (QCM-D) based sensor was developed to determine ApoE binding to LNPs, taking advantage of anti-PEG antibodies (manuscript in preparation). Figure 2 shows the binding isotherm of ApoE to LNPs as determined by QCM-D (see experimental details in the Methods section and the SI). No saturation of the LNP surface was obtained up to 2:1.06 wt % ApoE:mRNA (Figure 2A) since there was a continuous decrease in frequency with increased ApoE concentration in solution. A decrease in frequency shift directly translates to an increase in adsorbed wet mass. Moreover, the lack of spreading of the frequency overtones together with a minimal change in the energy dissipation (Figures 2A and SI4) suggests that ApoE adopts a rather compact and flat conformation on top of the LNP particles (Figure 2C). These conditions (no frequency spreading and low dissipation) enable the use of the Sauerbrey equation to determine the adsorbed mass.^42^ From the adsorbed mass and assuming a given packing of the LNPs on the sensor surface, the number of ApoE molecules per LNP can be estimated (Figure 2B). This number ranges between 250 and 340 ApoE molecules/LNP assuming either an LNP hexagonal or random packing. Based on geometrical considerations and assuming a hairpin-like configuration of the protein on the LNP surface^43^ (Figure 2C), the saturation of the LNP surface by adsorbed ApoE should occur around 180 molecules per LNP, which corresponds to 1:1 wt % ApoE:mRNA. Our results suggest that LNPs are able to bind more than a monolayer of protein or that ApoE does not adopt a hairpin conformation on the LNP surface.

In human blood serum, typical ApoE concentrations range on the order of 30–80 μg/mL. Here, LNPs were incubated with ApoE at the ratio of 1:1 wt % ApoE:mRNA (corresponding to 1:10 wt % ApoE:LNP components except mRNA), which is 10 to 100 times lower than the ratio found in blood assuming an mRNA dose of 0.3 mg/kg.^13,45^ Since ApoE is one of the most abundant proteins in LNP protein coronas, a high affinity between ApoE and LNPs is expected, as demonstrated by Figure 2 and by the fast kinetics of binding to LNPs (see inset in Figure 2A). Moreover, ApoE in human blood serum is bound to lipoproteins and not all is available to bind LNPs.

### Structural and Compositional Effects Induced by ApoE Binding to mRNA-LNPs

**Figure 3:**
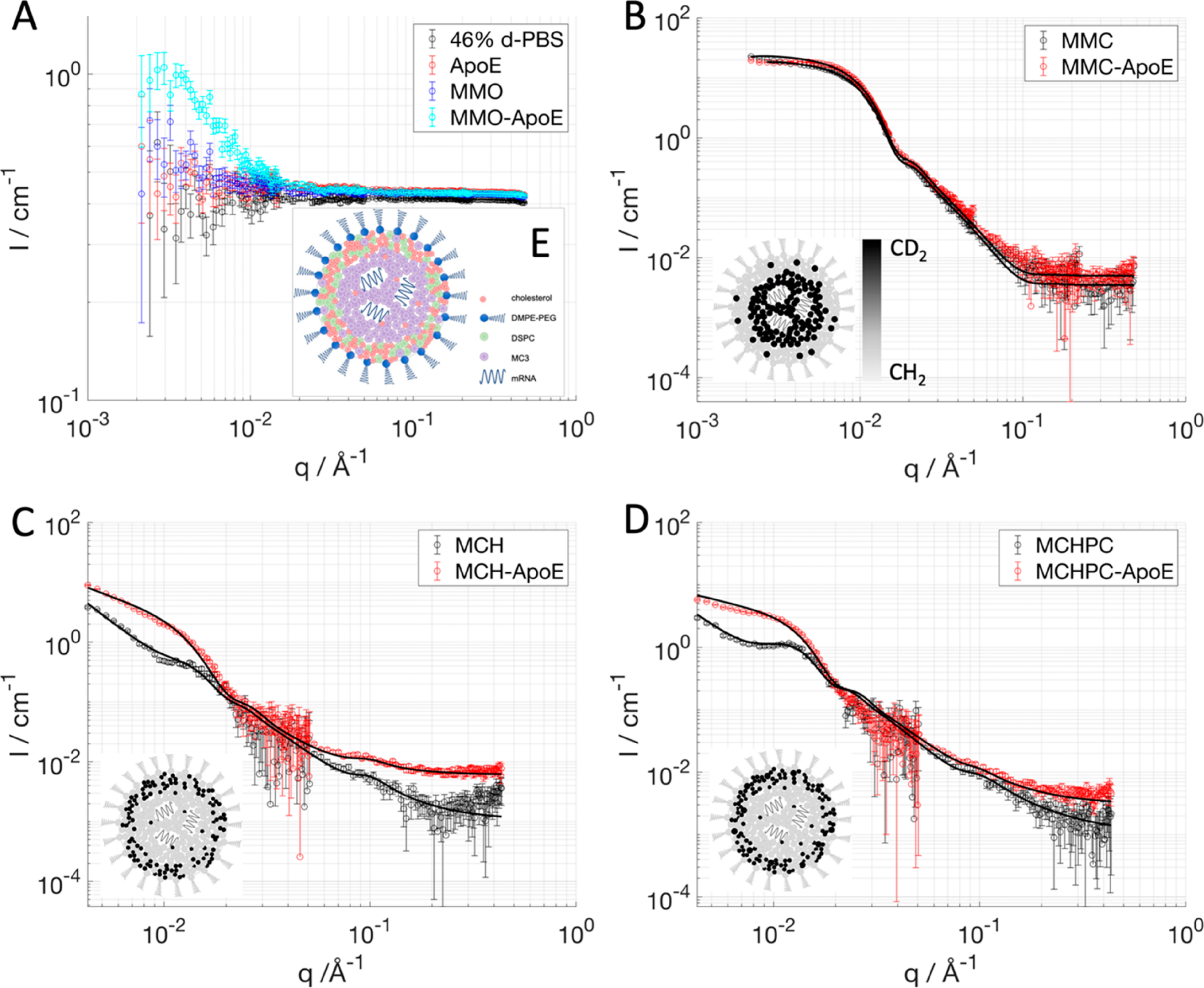
SANS data collected for the LNPs prepared with a mixture of deuterated and hydrogenated components (MMO) that allows the LNPs to be matched out in a buffer with 46% D_2_O content and to enhance the structural effect of ApoE incubation for 3 h: solvent containing 46% d-PBS (black symbols), ApoE (red symbols), MMO with (light blue symbols) and without (blue symbols) ApoE (A). LNPs prepared with dMC3 (MMC) and measured at 46% d-PBS with (red symbols) and without (black symbols) ApoE (B). SANS data for LNP prepared with 100% d-cholesterol (MCH) measured in 39% d-PBS with (red symbols) and without (black symbols) ApoE (C). LNP prepared with 100% d-cholesterol and 32% dDSPC (MCHPC) measured in 39% d-PBS with (red symbols) and without (black symbols) ApoE (D). Schematics of how the particle composition changes upon apolipoprotein binding: cholesterol moves toward the surface while MC3 partitions to the core (E). Solid lines are best fits to the experimental data. The nominal LNP composition is provided in Table 1. In the insets of panels B, C, and D the LNP schematics have the components colored according to their SLD values (*i*.*e*., deuterated components are black).

SANS data for MMO in 46% D_2_O based buffer and in the presence of ApoE are sensitive only to the composition and structure of the LNP. Indeed, 0.3 mg/mL ApoE was measured in 46% D_2_O based buffer, and the scattered intensity overlapped with the data collected for the solvent alone. This confirms that ApoE is invisible in this solvent condition (Figure 3A). Since the circulation time of LNPs in the body is approximately 3 h, MMO and ApoE were incubated in a 1:1 mixture (ApoE:mRNA wt %) at 25 °C for 3 h prior to SANS data acquisition. The SANS data after incubation with ApoE showed a clear deviation from matched out conditions (Figure 3A), which suggests a structural or compositional rearrangement of the particle. However, this does not allow to detail the changes in component distribution due to the complex particle composition and the poor contrast between shell and core.

So far, all the experiments described have involved the isoform ApoE3, being the most abundant allele and also having a neutral risk to develop atherosclerosis and Alzheimer’s disease (AD). However, we collected data for incubation of LNPs with ApoE4 (a proatherogenic variant and clinical marker for AD) and human serum albumin (HSA), respectively, to clarify how specific was the effect seen with ApoE3. SANS data for LNPs incubated with ApoE3 and ApoE4 show similar changes (Figure SI5), while the incubation of LNPs with HSA does not affect the structure of LNPs in a visible way (Figure SI6). Therefore, the structural and compositional effects hereby reported are not ApoE variant dependent and are specific to at least this type of apolipoprotein.

In order to clarify whether there is structural (*i*.*e*., domain formation at the LNP surface) or compositional (*i*.*e*., component redistribution between core and shell) change in LNPs upon ApoE binding, SANS data were collected with MCH, MCHPC, and MMC incubated with ApoE, all in solvent conditions that matched the SLD of ApoE within error (see Figure SI7, under the same experimental conditions). For all the samples, a difference in the scattering curve due to ApoE incubation was observed (Figure 3B–D), which enabled quantification of the compositional and structural changes occurring in the LNPs.

**Figure 4:**
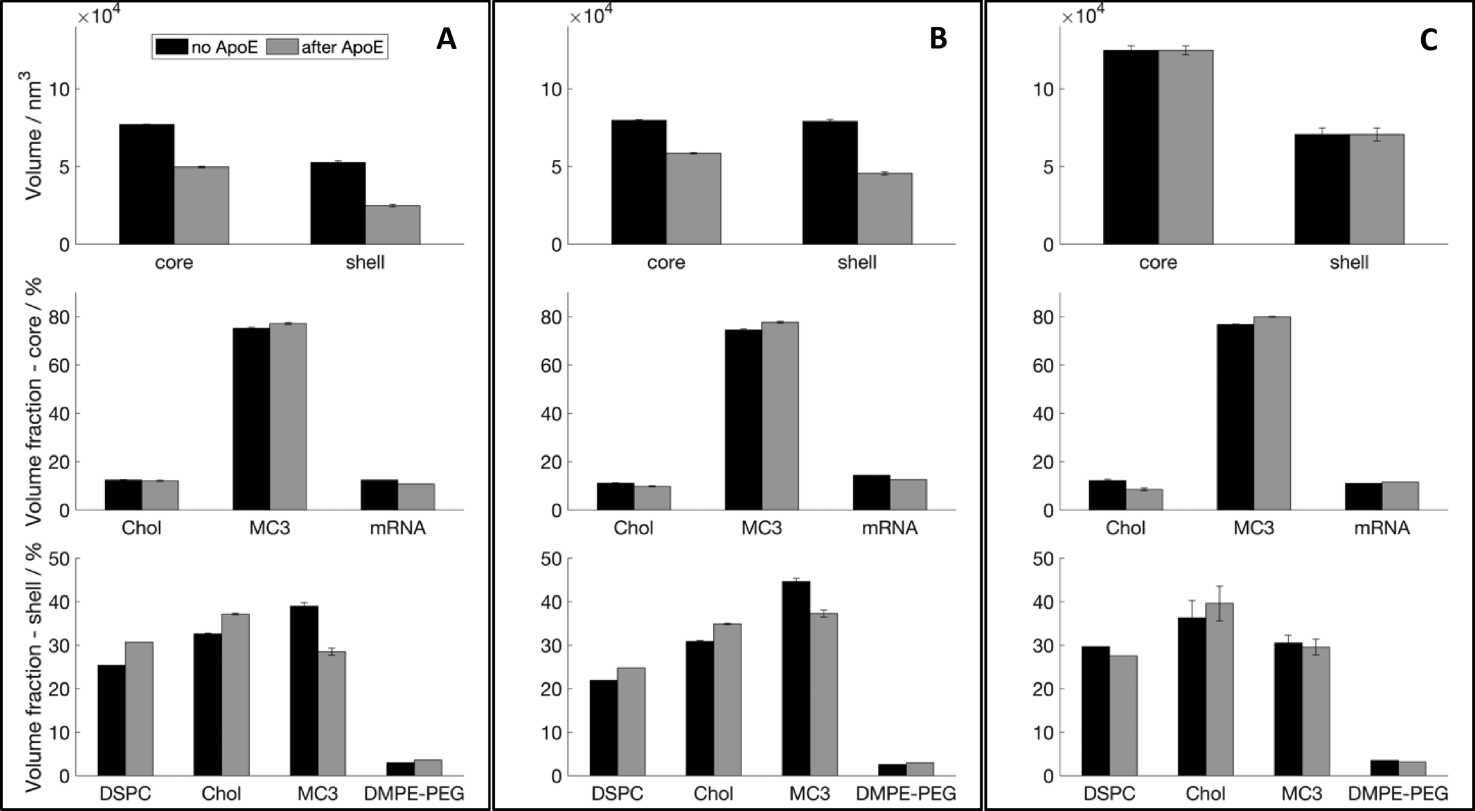
LNP volume and composition in the presence (gray) and absence of ApoE (black). The histograms in the top row show the volumes of shell and core calculated from the radius and thickness obtained fitting the SANS data respectively for MCH (A), MCHPC (B), and MMC (C). In the middle row, the histograms show the volume fractions for the LNP components present in the core when solvent is excluded: MCH (A), MCHPC (B), and MMC (C). In the bottom row, the histograms show the volume fractions for the LNP components present in the shell: MCH (A), MCHPC (B), and MMC (C). LNP samples were prepared according to Table 1. Changes in DSPC, cholesterol, and MC3 composition are significant.

All SANS curves collected after 3 h ApoE incubation show an increase of intensity at low *q* (Figure 3A–D). In the deuteration scheme for MCH and MCHPC (Figure 3C,D), the changes can be modeled with a slight decrease in size of the samples (Table SI3) and a larger decrease in the SLD of the core than the SLD of the shell for both samples (Table SI2). This suggests that the LNP compaction is accompanied by a redistribution of the LNP components from the shell to the core and/or *vice versa* (Figure 4). Assuming that ApoE does not remove components from the LNP particle, the observed changes in core and shell volume (Figure 4) and SLD (Table SI2) could be explained by MC3 being transferred from the LNP surface to the core, while the opposite takes place for cholesterol, resulting in an increased LNP surface cholesterol concentration (Figure 4 and Table SI6). Figure 4 shows the volume fractions normalized by either the shell or the core volume before and after ApoE incubation. Therefore, the net changes in the volume fraction of the core and in the shell are not expected to mass balance. Mass balance takes place if considering the number of molecules instead. The volume of the shell decreases to a larger extent than the core due to further thinning (Figure 4), which correlates with the observed decrease in MC3 surface concentration.

Previous structural analysis of mRNA-containing LNPs was performed at 25 °C;^20^ therefore we continued to use this temperature for comparative reasons. However, a more physiologically relevant temperature was tested (37 °C), giving no significant effect of temperature on LNP structure in the absence or presence of ApoE (Figures SI8–S10). Moreover, MCHPC and MCH in the presence and absence of ApoE were followed upon heating to 37 °C, then to 49 °C (in an attempt to melt the DSPC while keeping the protein active) and finally cooling back to 25 °C (Figure SI10). MCHPC incubation with ApoE was monitored as well for 21 h at 25 °C (Figure SI10D). Similar changes in the scattering curves took place regardless of incubation temperature. However, the protein incubation time with the LNP has dramatic effects on the scattering curves. Overall, this suggests that LNP structure and composition in physiologically relevant conditions should not differ significantly from the one determined here at 25 °C.

### ApoE Binding Results in Restructuring of Both LNP Surface and Core and Affects mRNA Encapsulation

For all LNPs exposed to ApoE samples, the modeled best fits are systematically higher than the SANS data for *q* < 0.005 Å^–1^ (Figure 3B–D), suggesting that the core–shell sphere model does not fully describe the particle form factor any longer even after accounting for component redistribution. The subtraction of the data collected with MMC from the MMC incubated with ApoE produces a clear peak at *q* = 0.006 Å^–1^ (see Figure SI11), which could indicate the domain formation at the surface with a distance proportional to 2π/*q* (∼100 nm). Similar effects are observed for the other data sets, where a failure of the core–shell model upon ApoE incubation seems evident.

**Figure 5:**
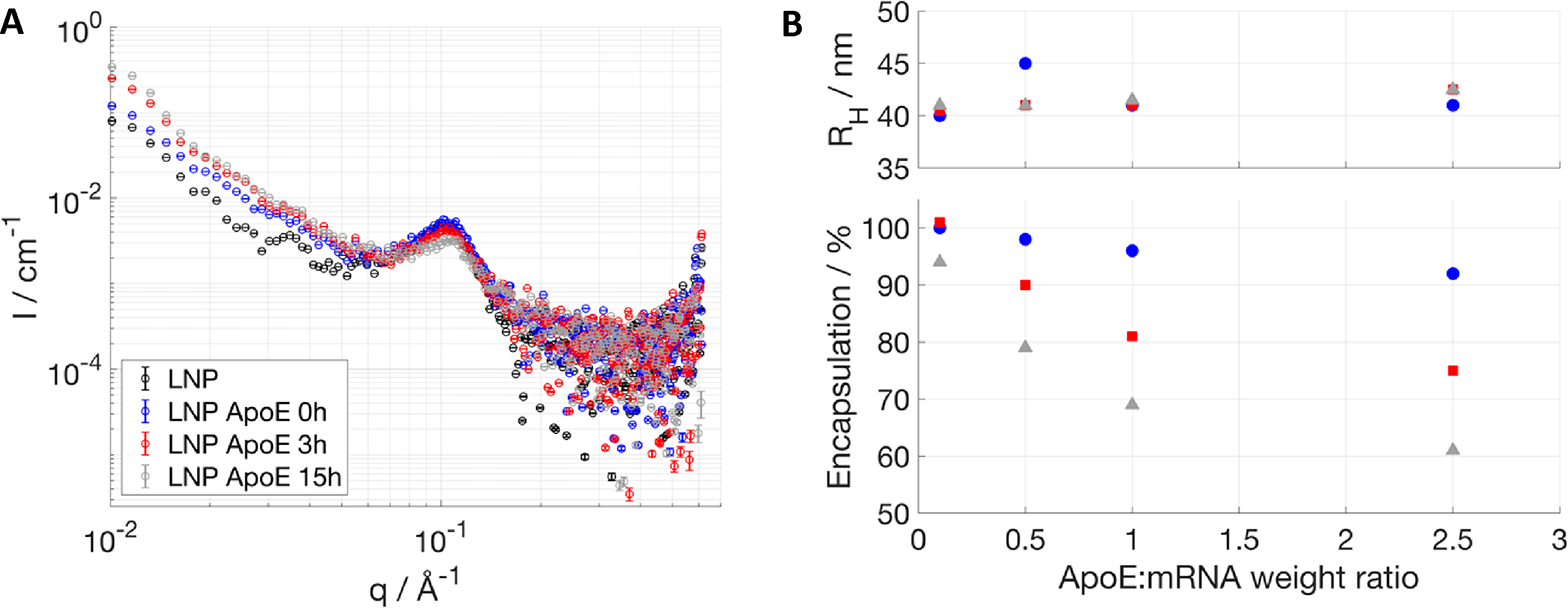
Stability of fully hydrogenated LNPs upon incubation with ApoE in terms of core structure measured by SAXS (A): SAXS patterns were measured at 1:1 wt % ApoE/mRNA at no ApoE (black), 0 h (blue), 3 h (red), and 15 h (gray) of incubation time. Size measured by DLS (B, top) and encapsulation efficiency (B, bottom). Increasing ApoE/mRNA weight ratios were used in B, and size and encapsulation efficiency were measured at day 0 (blue circles), 1 (red squares), and 3 (gray triangles) of incubation time. Error bars are almost always within the size of the symbols for SAXS, DLS, and encapsulation data.

To investigate the effect of ApoE incubation on the core structure, SAXS data were collected on LNPs with ApoE after mixing, after 3 and 15 h (Figure 5A). In this case, incubation with ApoE leads to a decrease in the peak intensity upon 3 h of incubation, which is then accompanied by a shift toward higher *q* upon 15 h of ApoE incubation besides further intensity decrease. This suggests a loss of order and a decrease of the *d*-spacing in the internal structure. The hydrodynamic radius (DLS) is not affected by ApoE addition (Figure 5B, top), in agreement with SANS that gave no or only minor effects in size (Table SI3). The same sample was tested for mRNA encapsulation (Figure 5B, bottom), and a drop of encapsulation was recorded after 1 day of incubation. This supports a change in the core structure allowing the mRNA to escape the LNP in time scales much longer compared to the ones required for the start of component redistribution as measured by SANS. This indicates that component redistribution and removal of cholesterol from the core occur, and, upon reaching a certain critical concentration, failure of the LNP core packing leads to RNA release.

While CIL in the core is key to pack the mRNA, the CIL at the LNP surface is thought to play a role in the endosomal escape. The data presented show that ApoE induces changes to the surface distribution of the lipids in the LNP and that the LNPs decrease in size. Earlier results in our group suggest that ApoE selectively interacts with lipids rather than cholesterol when exposing ApoE to supported lipid bilayers made of cholesterol and phospholipids (manuscript in preparation). Even though our data do not demonstrate lipid removal from LNPs by ApoE, a possible explanation for the shell enrichment in cholesterol is that ApoE selectively removes lipids and not cholesterol from the shell. Thus, it is possible that some of the CIL found in the liver is not bound to LNPs but to lipoproteins in the blood. However, the binding of the protein to the LNPs takes 10–20 min to reach equilibrium (Figure 2A inset), while the structural rearrangement is a process on a time scale of hours. Within 2 h following intravenous administration, only about 20% of the initial injected dose of siRNA-LNP formulated with DMPE-PEG is found in the blood, while more than 60% is in the liver.^11^ Therefore, the binding of ApoE may trigger the recognition in the liver by LDL receptors within 20 min of administration, while longer time scales are needed for component redistribution by ApoE.

Finally, the exact role of cholesterol in LNP endosomal escape is unknown. It is well known that cholesterol is needed for both endocytosis and endosomal escape for a range of viruses^46,47^ and for lipoplexes.^48^ However, other reports suggest that late endosomal/lysozomal cholesterol accumulation in the host protects against the endosomal escape for influenza A virus.^49^ Interestingly, a very recent publication has shown that the replacement of cholesterol by cholesterol analogues in LNPs dramatically improves the transfection efficiency, probably due to steady endosomal escape.^18^ The cholesterol analogue containing LNPs had an irregular surface,^18^ which may suggest that surface domains could facilitate the endosomal escape. Moreover, the substitution with cholesterol analogues modulated NPC1/2 activity (a protein mediating the escape of cholesterol from late endosomes to the cytosol),^50^ reduced LNP efflux, and improved intracellular availability and mRNA delivery.^18^ Thus, we can hypothesize that not only the LNP surface composition but also the surface nanostructure is important. In this work, we show that ApoE binding leads to an increased cholesterol concentration in the LNP surface, which seems to be accompanied by nanodomain formation.

## Conclusions

mRNA-LNPs with the composition MC3:DSPC:Chol:DMPE-PEG 50:10:38.5:1.5 mol % were confirmed to have a core–shell structure; a partitioning of cholesterol toward the surface was demonstrated, which is between 2 and 4 times as concentrated than in the core, while the MC3 is almost twice as concentrated in the core than in the shell of the LNPs. Even though some variation in size is found among different LNP batches, the composition across shell and core remains constant.

Once LNPs come in contact with ApoE, not only does protein adsorption at the particle surface occur, but there is a rearrangement of both the surface and core structures. These changes are accompanied by a redistribution of the lipid components in the LNP. In the literature, it is well known that proteins in serum bind to nanoparticles, and the data presented here demonstrate that protein absorption can affect the internal structure and component distribution of lipid-based nanoparticles. The effect of protein on the LNP structure might be irreversible; hence it is important for an understanding of the fate of LNPs after cellular uptake, and the ability to escape the endosome is key for the protein expression.

## Materials and Methods

### Materials

The lipids used for LNP formulations were *O*-(*Z*,*Z*,*Z*,*Z*-heptatriaconta-6,9,26,29-tetraem-19-yl)-4-(*N*,*N*-dimethylamino)butanoate (DLin-MC3-DMA, AstraZeneca), cholesterol (Sigma-Aldrich), 1,2-distearoyl-*sn*-glycero-3-phosphocholine (DSPC, CordenPharma), d83-DSPC (Avanti Polar Lipids), and 1,2-dimyristoyl-*sn*-glycero-3-phosphoethanolamine-*N*-[methoxy(polyethylene glycol)-2000] (DMPE-PEG2000, NOF Corporation)). All LNP samples contained CleanCap enhanced green fluorescent protein (eGFP) mRNA (5-methoxyuridine) (TriLink Biotechnologies). Human ApoE3 was purchased from Sigma-Aldrich and used without further purification (Product number SRP4696, purity >90% SDS-PAGE and HPLC). Alternatively, human ApoE3 and ApoE4 were produced according to ref (20) with some minor modifications of the protocol: 6 M urea for denaturation was used, and refolding was done in phosphate buffer instead of Tris. Gold-coated QCM-D sensors were purchased from Biolin Scientific. PEG-thiol and biotin-PEG thiol were purchased from Polypure AS (product number 10156-0795 and 41156-1095). Streptavidin from *Streptomyces avidin* and bovine serum albumin were purchased from Sigma-Aldrich (product number S4762 and A8806). Anti-polyethylene glycol antibody [PEG-B-47b] conjugated to biotin was purchased from Abcam (product number ab53449). Phosphate buffer saline (PBS, 4 mM, 155 mM NaCl pH 7.13H/6.9D at D22, 10 mM NaCl 150 mM pH 7.4 at KWS2) was prepared with potassium phosphate monobasic, sodium phosphate dibasic, and sodium chloride. Millipore water or deuterated water (Sigma-Aldrich 151882 purity 99.9% at D22, Armar isotopes 99.9% at KWS-2) was used for buffers and sample dilution.

### Deuterated Compounds

Deuterated MC3 (D_62_ 99.3%, dMC3) was synthesized and purified by AstraZeneca; the synthesis was performed according to the protocol described by Jayaraman and co-workers,^14^ replacing linoleic acid ethyl ester with the corresponding deuterated compound. The deuterated starting material, linoleic acid (18:2), ethyl ester (D_31_ 98%), was purchased from Cambridge Isotope Laboratories Inc., Andover, MA, USA.

Deuterated cholesterol^38,51^ (average 89% D) was produced by the Deuteration Laboratory within ILL’s Life Science Group^52^ according to the protocol described by Waldie and co-workers;^38^ detailed analysis and NMR and MS spectra can be found in ref (38).

Deuterated cholesterol^53^ (average 87% D) was produced by ANSTO’s National Deuteration Facility using *Saccharomyces cerevisiae* strain RH6829.^53^ In this work, yeast growth medium comprised 0.7% yeast nitrogen base, 0.5% yeast extract, 1.25% glucose, and 30 mg/L each of uracil and l-leucine per liter in deuterium oxide (D_2_O, 99.8% D atomic purity) (Sigma-Aldrich). A single colony was picked from an agar plate and inoculated into 50% D_2_O (1:1 D_2_O/H_2_O) yeast growth medium and incubated at 30 °C while shaking at 200 rpm. After 2 days, 1 mL of turbid culture was inoculated into 50 mL of 100% D_2_O yeast growth medium and incubated as above. This seed culture was inoculated into 3 L of 100% D_2_O yeast growth medium and cultivated in a Minifors 2 bioreactor (Infors, Switzerland) until a stationary phase was observed (indicated by a rise in dissolved oxygen and pH signals). Cells were harvested by centrifugation and saponified in a solution of 15% KOH, 71% methanol, and 0.125% pyrogallol (w/v). After refluxing for 2 h at 90 °C, the mixture was extracted three times with *n*-hexane. The solvent was evaporated, and the residue was purified by silica column chromatography, prepared with *n*-hexane. Deuterated cholesterol (average 87% D) was isolated by eluting with *n*-hexane/ethyl acetate (9:1 v/v) and identified in fractions by thin layer chromatography (TLC) using Kieselgel silica gel 60 F254 aluminum sheets (Merck). The % deuterium incorporation for nonlabile protons in the molecule was calculated by averaging electrospray ionization–mass spectrometry (ESI-MS) peak areas for the different deuterated isotopomers (AB Sciex). NMR spectra were recorded on a Bruker Avance III 400 MHz spectrometer at 298 K, equipped with a 5 mm PABBO BB H/D z-gradient probe. Spectra were referenced to residual deuterated solvent. ^2^H and ^13^C NMR with proton as well as proton and deuterium nuclei decoupled were recorded as detailed in a previous publication.^54^^13^C resonances attached to deuterium appear as multiplets when only the proton nucleus is decoupled ^13^C {^1^H} and resolved to singlets when both proton and deuterium nuclei are decoupled (*i*.*e*., ^13^C {^1^H,^2^H}). The level of deuterium labeling at some specific sites in the molecule was calculated using ^13^C {^1^H,^2^H} NMR according to the published method by Darwish *etal*.^54^ Detailed analysis and NMR and MS spectra of deuterated cholesterol (average 87% D) can be found in the SI.

### LNP Preparation and Characterization

LNPs were prepared using a NanoAssemblr microfluidic instrument (Precision NanoSystems Inc.). Lipid stocks were prepared in ethanol and mixed at suitable molar ratios, while the mRNA was diluted in 50 mM citrate buffer pH 3. The lipid composition of the LNP in this work is MC3:DSPC:Chol:PEG-DMPE 50:10:38.5:1.5 mol %. mRNA was added to have a nucleotide to MC3 ratio of 1:3. The mRNA and lipids were mixed in a 3:1 volume ratio at a 12 mL/min speed. LNPs were dialyzed overnight in phosphate buffer saline using Slide-A-Lyzer G2 dialysis cassettes with a molecular weight cutoff of 10 K (Thermo Scientific). Particle size was characterized through DLS with a Zetasizer Nano ZS (Malvern Instruments Ltd.), and encapsulation and mRNA concentration were measured using the Ribogreen assay and found to be above 95% for all samples (see Tables 2 and SI1). Ribogreen is a fluorescent dye that has enhanced emission when it binds to nucleic acids. This dye is typically added to the samples before and after solubilization of LNPs in a detergent solution (2% Triton TX-100). The free mRNA in solution is compared with the total mRNA concentration after solubilization of the LNPs to estimate the encapsulation percentage.

### Binding Isotherm for ApoE to LNPs

The isotherm for ApoE binding to LNPs was obtained by addition of ApoE on a precoated LNP QCM-D gold sensor. A full description of the sensor performance will be reported in an upcoming publication (manuscript in preparation). The protocol for LNP immobilization on the sensor is described in the SI. To determine the binding of ApoE to precoated LNP sensors, a QCM-D analyzer (Q-sense) with four independent flow modules was used. ApoE stock solution (0.5 mg/mL) was diluted to 0.5, 2.5, 5, and 12.5 μg/mL. After rinsing the immobilized LNPs with PBS, 1 mL of each ApoE dilution was injected to a sensor and left for about 10 min, then rinsed with PBS. The frequency value obtained after PBS rinsing was compared to the value recorded after immobilization of LNP rinsing.

The frequency shifts and dissipation changes upon protein addition were analyzed by the Sauerbrey equation,^42^ and the corresponding wet mass adsorbed was obtained.

### Small-Angle Neutron Scattering Experiments

For the SANS study, the contrast matching approach was exploited.^24−26,55^ In sample MCH (see Table 1), all cholesterol was substituted with d-cholesterol (average 89% D, SLD 6.5 × 10^–6^ Å^–2^)^38^ to highlight its localization and partitioning. In sample MCHPC, 32% mol of d83-DSPC and 100% mol d-cholesterol (average 89% D) was used instead as an initial step to match out the particle in 39% D_2_O (see Figure SI1). In the sample MMC, 100% of d62-MC3 (SLD 5.1 × 10^–6^ Å^–2^) was used. Finally, a matched out LNP sample (MMO) was obtained mixing deuterated and hydrogenated components in appropriate molar ratios: 37% d83-DSPC, 42% d62-MC3, and 42% d-cholesterol (average 87% D). The latter formulation was based on volume fraction determination based on the analysis of SANS data collected for the 100% d-cholesterol (average 89% D) sample with the aim to match out completely the core with the shell and the solvent, as it was demonstrated to be possible by Heberle and co-workers^56^ for liposomes. These four samples were characterized with DLS, and they reported a similar hydrodynamic radius (⟨*Z*⟩, intensity weighted) and polydispersity index, PDI (see Table SI1).

Samples MCH and MCHPC were measured on D22 at ILL.^57^ Briefly, data were collected in the *q*-range 0.0028–0.46 Å^–1^ using a 6 Å wavelength, two detector distances (2 and 17.6 m), and two collimation distances (2.8 and 17.6 m). Data were reduced and scaled for absolute intensity according to standard procedures using GRASP version 8.16l.^58^ Samples MMC and MMO were measured on KWS-2^59,60^ at FRM-II. Data were collected in the *q*-range 0.001–0.48 Å^–1^ using the four detector distances (20, 14, 4, and 2 m), three collimation distances (20, 14, and 4 m), and two wavelengths: 10 Å for the longest sample–detector distance and 4.66 Å for the rest; data were reduced and scaled for absolute intensity according to standard procedures by qtiKWS software^61^ provided by JCNS. All the data were merged and background subtracted using the macro on Igor Pro.^62^ SANS curves were collected for all solvent contrasts in the same conditions as the samples and used as background. Samples were loaded in Hellma cells (1 mm path length) and then placed in a temperature-controlled sample changer, and temperature was set to 25 °C. Samples MCH and MCHPC were measured once equilibrated at *T* = 37 and 49 °C and then after cooling at 25 °C.

### SANS Contrast Matching

The LNP samples were diluted at given solvent ratios of D_2_O/H_2_O to a final mRNA concentration of 0.3 mg/mL in order to highlight different parts of the LNP. To follow the effect of ApoE on the LNP structure, the ApoE and LNP were mixed, and the sample was measured at different time points (D22) or only after 3 h incubation (KWS-2).

### SANS Data Analysis and Interpretation

SANS data were first analyzed to obtain the pair distance distribution function *p*(*r*) (GIFT software),^63^ and from the *p*(*r*) the radial density profile, *d*(*r*) (DECON software),^64^ was obtained. The *p*(*r*) and the *d*(*r*) helped in the selection of the analytical model to be used in the fitting (*p*(*r*) and *d*(*r*) in the SI, Figure SI3). SasView software was used for the analysis of the data. A core–shell sphere model^27^ with a polydispersity of the core radius fixed to a suitable value (0.1 for MMC and 0.12 for MCH and MCHPC) was applied to all the SANS data separately, and then the simultaneous fit was implemented. Data collected with the same LNP formulation but different solvent D_2_O content were simultaneously fitted to a core–shell sphere model; details on the constraints are given in the Results section. The free parameters were the core radius, the shell thickness, the shell SLD, and the core SLD, while the remaining parameters were kept constant to known or estimated values. Some of the samples (MCH and MCHPC) showed a clear peak around *q* ≈ 0.1 Å^–1^ arising from the internal structure; this has been included in the model using a broad peak model in addition to the core–shell sphere model. For these samples, the combined model was applied to each curve separately, keeping constant all the parameters previously optimized for the core–shell sphere model and the structural parameters of the broad peak model, while the intensity (*i*.*e*., contrasts)-related parameters where allowed to vary (see the SI for details). From the fitted SLD values of core and shell and their volumes, the volume fractions and molar fractions for each component in the shell and the core were determined (further details can be found in the SI).

### Small-Angle X-ray Scattering Experiments

SAXS experiments were performed using a Mat:Nordic instrument from SAXSLAB Aps. This instrument is equipped with a Rigaku 003+ high-brilliance microfocus Cu-radiation source and a 300 K Pilatus detector. The setup employed for the measurements covered the *q* range of 0.011 < *q* (Å^–1^) < 0.68. The measurements of LNPs were carried out with reusable quartz capillaries of 1.5 mm diameter, which were placed in a thermostated block connected to a circulating water bath to maintain the temperature at 25 °C. The concentration of the samples for these measurements was 0.5 mg/mL of mRNA and a 1:1 mRNA:ApoE weight ratio. Each sample was measured for 20 min. The data presented are background subtracted, where the background corresponds to the buffer measured in the same capillary.

## Supporting Information Available

DLS and encapsulation efficiency for all LNP samples, matching conditions for MCHPC, SANS data and model fit of MMO, details on SANS fitting procedure and compositional analysis, pair distance distribution function and radial density profile, SLD values resulting from SANS model fitting, determination of volume fraction from SLD and then molar fraction, protocol for LNP immobilization on gold sensors for QCM-D and raw data for ApoE binding to immobilized LNPs, SANS data for LNP incubation with ApoE4 and HSA, SANS data of ApoE in contrast match condition, SANS data for MCH and MCHPC upon heating, SANS data of MCH and MCHPC upon incubation with ApoE and heating, scattering intensity resulting from subtraction of SANS data collected with MMC from MMC incubated for 3 h with ApoE, details on deuterated cholesterol (PDF)
