# Supplementary material for: Apolipoprotein E Binding Drives Structural and Compositional Rearrangement of mRNA-Containing Lipid Nanoparticles
Source: ACS Nano. 2021 Mar 23;15(4):6709–22. doi: 10.1021/acsnano.0c10064 (PMC8155318; doi:10.1021/acsnano.0c10064)
Supplement: Supplementary file 1 — nn0c10064_si_001.pdf [file nn0c10064_si_001.pdf]

Supplementary Information to

Apolipoprotein E Binding Drives Structural and Compositional Rearrangement of mRNA Containing Lipid Nanoparticles

Federica Sebastiani[1], Marianna Yanez Arteta[2*], Michael Lerche[2], Lionel Porcar[3], Christian Lang[4], Ryan A. Bragg[5], Charles S. Elmore[6], Venkata R. Krishnamurthy[7], Robert A. Russell[8], Tamim Darwish[8], Harald Pichler[9,10], Sarah Waldie[1,11,12], Martine Moulin[11,12], Michael Haertlein[11,12], V. Trevor Forsyth[11,12,13], Lennart Lindfors[2] and Marité Cárdenas[1*]*

[1]Biofilms - Research Center for Biointerfaces and Department of Biomedical Science, Faculty of Health and Society, Malmö University, 20506 Malmö, Sweden. [2]Advanced Drug Delivery, Pharmaceutical Sciences, R&D, AstraZeneca, 431 83 Gothenburg, Sweden [3]Large Scale Structures, Institut Laue Langevin, Grenoble F-38042, France. [4]Forschungszentrum Jülich GmbH, Jülich Centre for Neutron Science JCNS, Outstation at Heinz Maier-Leibnitz Zentrum, Lichtenbergstraße 1, 85748 Garching, Germany. [5]Early Chemical Development, Pharmaceutical Sciences, R&D, AstraZeneca, SK10 4TG Cambridge, UK. [6]Early Chemical Development, Pharmaceutical Sciences, R&D, AstraZeneca, 431 83 Gothenburg, Sweden. [7]Advanced Drug Delivery, Pharmaceutical Sciences, R&D, AstraZeneca, CB2 0AA Boston, USA. [8]National Deuteration Facility (NDF), Australian Nuclear Science and Technology Organisation

(ANSTO), Lucas Heights, 2232 Sydney, NSW, Australia. [9]Austrian Centre of Industrial Biotechnology, Petersgasse 14, 8010, Graz, Austria. [10]Graz University of Technology, Institute of Molecular Biotechnology, NAWI Graz, BioTechMed Graz, Petersgasse 14, 8010, Graz, Austria. [11]Life Sciences Group, Institut Laue Langevin, Grenoble F-38042, France. [12]Partnership for Structural Biology (PSB), Grenoble F-38042, France. [13]Faculty of Natural Sciences, Keele University, Staffordshire, ST5 5BG, UK.

Corresponding authors*:
Federica Sebastiani federica.sebastiani@mau.se
Marianna Yanez Arteta mariann.yanezarteta@astrazeneca.com
Marité Cárdenas marite.cardenas@mau.se

LNP characterization, Fitting Procedure and Compositional Analysis

All LNP samples were characterised prior to SANS experiments both for size with DLS and encapsulation efficiency with Ribogreen assay.

Table SI1. DLS- diameter intensity-weight <Z>, number-weight <N> and volume-weight <V> averages as well as encapsulation efficiency (EE) for the various LNP samples used. Errors on EE are typically below 1%

Sample	<Z> (nm)	PDI	<N> (nm)	<V> (nm)	EE* (%)
MCH	85 ± 1	0.13 ± 0.01	60 ± 4	75 ± 2	95
MCHPC	87 ± 1	0.07 ± 0.01	68 ± 4	80 ± 3	97
MMO	81 ± 1	0.05 ± 0.03	65 ± 3	75 ± 2	97
MMC	86 ± 1	0.020 ± 0.003	70 ± 2	80 ± 1	98

* Each sample was measured in duplicate and concentrations were compared to standard curves per experiment.

MCHPC was initially designed to be matched out close to ApoE matching conditions (43% d-PBS), and in Figure SI1 the contrast matching plot is shown. The composition was not optimal and scattering intensity was minimized but never went to zero.

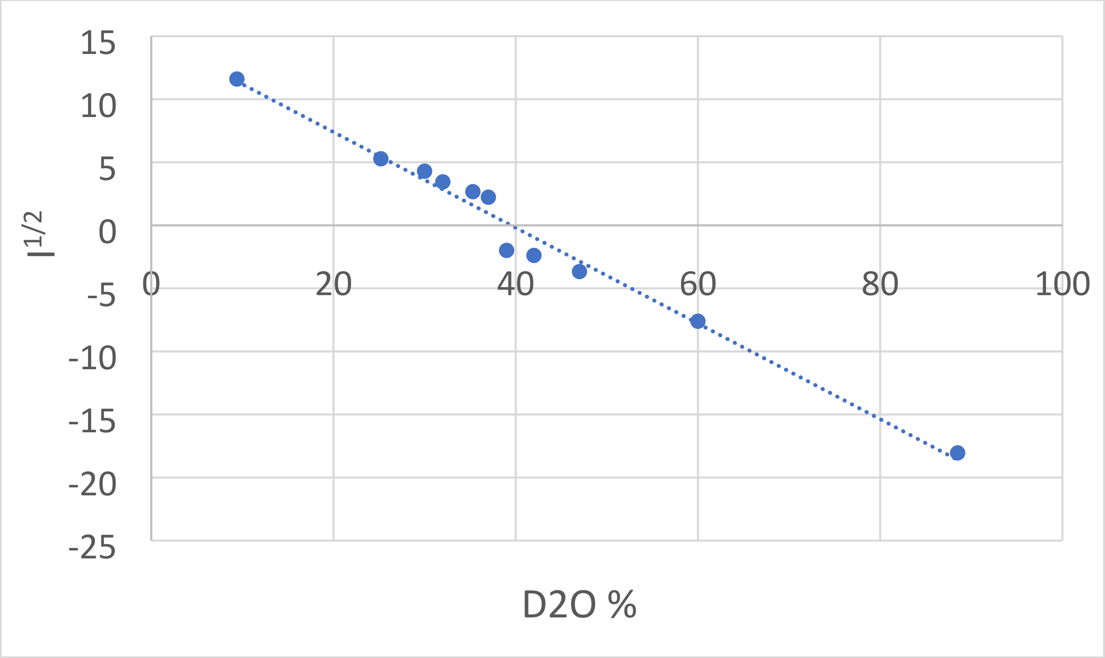

Figure SI1. Neutron contrast matching plot of a squared intensity (average over low q) against D_2O content in the H-D buffer. The contrast matching point ($I^{1/2}$ close to zero) is found at 39% D_2O.

In the following, the procedure to analyze the SANS data collected on the different LNP samples is explained in detail.

The sample MMO was described by a sphere model (Fig. SI2), since the deuteration scheme led to an indistinguishable core-shell structure.

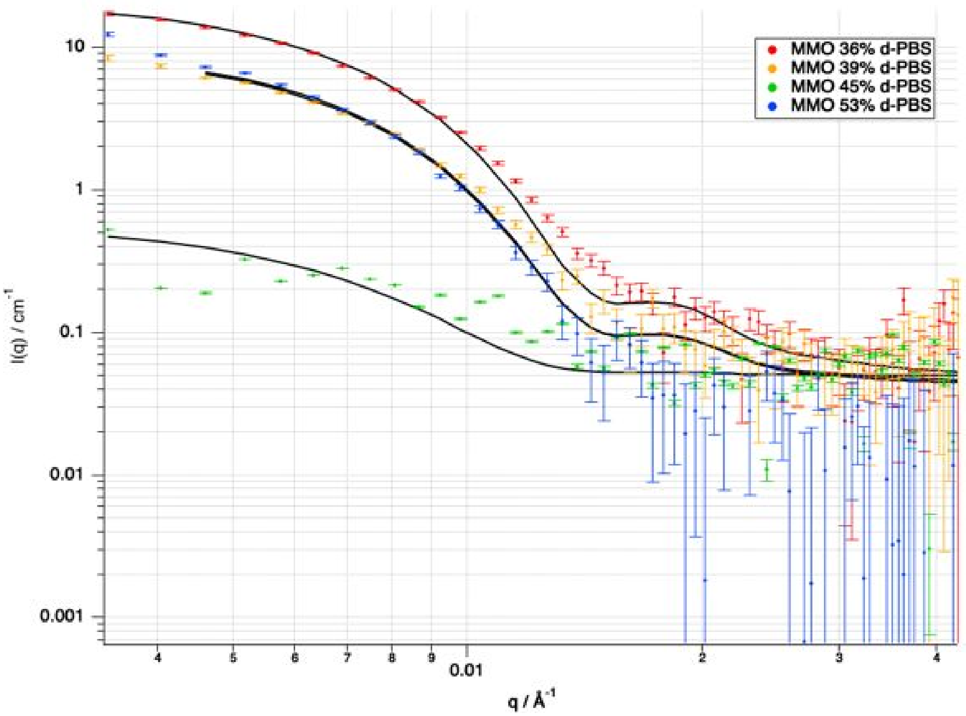

Figure SI2. SANS curves recorded for MMO in different solvent contrasts (% d-PBS) and sphere model fit (black solid line).

For all the LNP samples, except MMO, a preliminary analysis of the data was carried out: the pair distance distribution function (p(r)) and the density profile (d(r)) were obtained from the SANS curves in order to support the choice of model for the fitting. GIFT[1] and DECON[2] were used respectively to determine the pair distance distribution function and the density profile as a function of particle radius. Figure SI3 shows the p(r) and d(r) for sample MMC in different solvent contrasts, and comparing the different curves it is clear the indication toward a core shell structure.

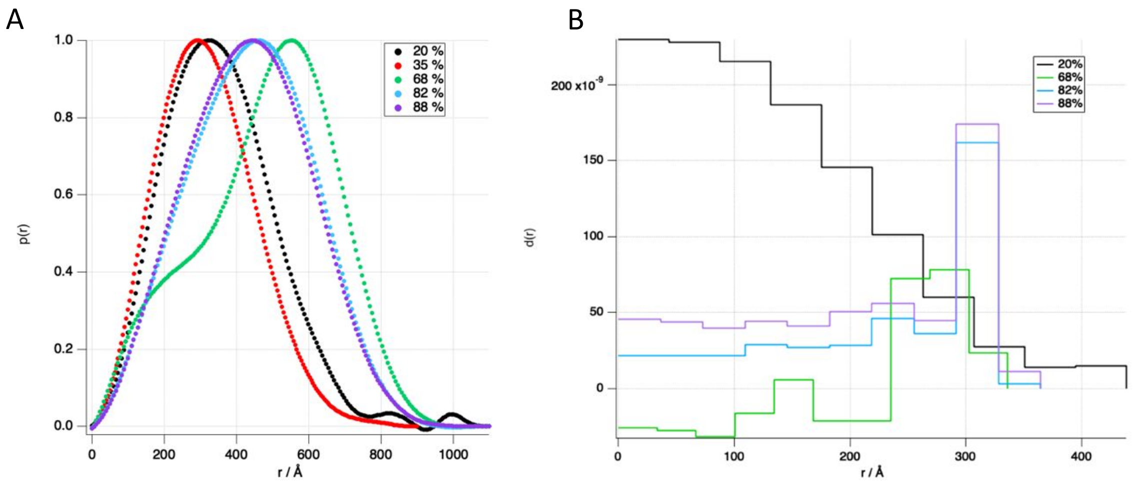

Figure SI3. Normalized p(r) of MMC (A)

and density profile as a function of radius of MMC (B).

For MCH and MCHPC, the fitting procedure was a two-step process, however the first step was common to MMC as well. The first step was a simultaneous fit using the core shell sphere model applied to the 4 (or 5) curves. The core radius, shell thickness and shell scattering length density (SLD) were constrained to be the same amongst the different solvent contrasts, while the core SLD was allowed to vary accounting for solvent in the core (SLDcore=vfsol×SLDsol+(1-vfsol)×SLDdry core). Since the SANS curves for MCH and MCHPC showed a clear peak at q ~0.1 Å-1, the final model was a sum of core shell sphere and broad peak models to better describe the data in the q range above 0.05 Å-1. This broad peak arises from the internal structure in the core of the LNPs. The combined model was applied to each curve separately, keeping constant all the parameters previously optimized for the core shell sphere model and the structural parameters of the broad peak model while the intensity (*i.e.* contrasts) related parameters where allowed to vary.

The scale factor was fixed to the volume fraction (determined by the sample concentration), the background value was optimized for each curve and the solvent SLD was set as calculated from the mixing ratios of H_2O/D_2O.

From the core SLD and shell SLD (Table SI2), the volume fraction of each component can be determined. For each lipid component in the LNP, the molecules partition between shell and core with the distribution being constrained by the molecular volume and the volume of shell and core. The use of molecules with different SLDs allows to refine the partitioning, which is optimized to obtain estimated SLDs that matches the SLD values from the fitting (Table SI2). The broad peak model accounts for the internal core structure, and becomes relevant at low q as well when overall intensity of the scattering curve is low (*i.e.* MCH and MCHPC in 39% D_2O based buffer) since MC3 has a SLD of 0.08×10^{-6}Å^{-2} and it has increasing contrast when d-PBS % > 50%.

Table SI2. SLD values resulting from the fit of SANS curves collected with LNPs with and without ApoE incubation for 3 hours.

	Prior ApoE incubation		Upon ApoE incubation	
	SLD dry core /10^{-6}Å^{-2}	SLD shell/10^{-6}Å^{-2}	SLD dry core /10^{-6}Å^{-2}	SLD shell /10^{-6}Å^{-2}
MCH	1.79 ± 0.01	2.73 ± 0.01	1.08 ± 0.01	2.50 ± 0.01
MCHPC	1.49 ± 0.01	2.93 ± 0.01	0.82 ± 0.01	2.83 ± 0.01
MMC	4.37 ± 0.01	1.7 ± 0.1	4.54 ± 0.11	1.63 ± 0.13
MMO*	2.58 ± 0.01	NA	2.75 (2.49)** ± 0.01	NA

* The sphere model was used for modelling MMO and hence SLD dry core corresponds to the SLD of the sphere, since there is a lack of contrast between shell and core in the experimental conditions used.

** The increase in SANS intensity (I(q)) upon ApoE incubation for MMO can be described by both an increase or decrease of SLD dry core, since the I(q) is proportional to the square of the $SLD_{dry\ core}$-$SLD_{solvent}$ and we have only measured MMO+ApoE in one contrast.

Table SI3. Net changes in structural parameters occurring upon 3h ApoE incubation. For MMC and MMO the structural parameters were kept fixed when fitting the SANS curve collected after ApoE incubation.

Net change	MCH+ApoE	MCHPC+ApoE	MMC+ApoE	MMO+ApoE
Core Radius /nm	-3.6 ± 0.3	-2.6 ± 0.3	NA	NA
Shell Thickness /nm	-1.7 ± 0.2	-1.8 ± 0.2	NA	NA
Total Radius /nm	-5.3 ± 0.5	-4.4 ± 0.4	NA	NA

Calculations: from SLD to volume fraction and then to molar fraction

From SLD to volume fraction

$$v_{f,x,i} = {}^{V_i}\!/_{V_{x,LNP}}$$

i is any of the LNP components included the solvent

x is either shell or core

$$SLD_{shell} = \sum_i v_{f,shell,i} SLD_i$$

$$SLD_{core} = \sum_i v_{f,core,i} SLD_i$$

$$SLD_{core} = v_{f,solv} SLD_{solv} + (1 - v_{f,solv}) SLD_{core\_dry}$$

From volume fraction to molar fraction

Molar fraction of component x $f_{M,x} = \frac{N_{M,x}}{N_{tot}}$

Volume fraction of component x $f_{v,x} = \frac{V_{M,x}}{V_{tot}}$

Number of moles of component x $N_{M,x} = \frac{V_{M,x}}{V_{mol,x}}$

Total number of moles $N_{tot} = \sum_i \frac{V_{M,i}}{V_{mol,i}} = \sum_i \frac{f_{v,i} V_{tot}}{V_{mol,i}} = V_{tot} \sum_i \frac{f_{v,i}}{V_{mol,i}}$

Molar fraction of component x $f_{M,x} = \frac{V_{M,x}}{V_{mol,x}} \frac{1}{V_{tot}} \frac{1}{\sum_i \frac{f_{v,i}}{V_{mol,i}}} = f_{v,x} \frac{1}{V_{mol,x}} \frac{1}{\sum_i \frac{f_{v,i}}{V_{mol,i}}}$

Error determination on volume fractions and molar fractions

Errors on volume fractions were derived from error on fitted SLD for shell and core separately *e.g.* $dSLD = SLD_{chol} * dvf_{chol} + SLD_{MC3} * dvf_{MC3}$. The relative errors on the molar fractions were obtained by summing the relative error on the corresponding volume fraction and the one on the term $\sum_i \frac{f_{v,i}}{V_{mol,i}}$.

Table SI4. Molar fractions estimated from the fitted SLD using the core shell model. Errors are estimated from the errors on volume fractions.

	Shell molar fractions %				Core 'dry' molar fractions %		
Sample	DSPC	Chol	MC3	DMPE-PEG	Chol	MC3	mRNA
MCH	18.0 ± 0.2	50.0 ± 0.8	29.0 ± 0.9	3.00 ± 0.03	25.0 ± 0.6	75 ± 1	0.0300 ± 0.0002
MCHPC	16.0 ± 0.2	48.0 ± 0.8	34.0 ± 0.9	2.00 ± 0.02	23 ± 0.6	77 ± 1	0.0400 ± 0.0003
MMC	21.0 ± 1.5	54 ± 10	22 ± 3	3.0 ± 0.2	24.0 ± 1.3	76 ± 1	0.0300 ± 0.0004
average	18 ± 2	51 ± 3	28 ± 6	2.7 ± 0.6	24 ± 1	76 ± 1	$3.3 \pm 0.6\ 10^{-2}$

Table SI5. Molar compositions in % of the LNP compared to the mixing ratios (last row) used to formulate.

	Molar composition determined by SANS				
	DSPC	Chol	MC3	DMPE-PEG	mRNA
MCH	9.0 ± 0.2	37.5 ± 1.3	52 ± 2	1.50 ± 0.04	0.0150±0.0004
MCHPC	8.0 ± 0.2	35.5 ± 1.3	56 ± 2	1.00 ± 0.03	0.0200±0.0005
MMC	10.5 ±1.6	39 ± 9	49 ± 9	1.5 ± 0.2	0.0150±0.0014
Mixing ratios	10	38.5	50	1.5	0.015

Table SI6. Molar fractions estimated from the fitted SLD using the core shell model for LNP after incubation with ApoE3 for 3 hours.

	Shell molar fractions				Core 'dry' molar fractions		
Sample	DSPC	Chol	MC3	DMPE-PEG	Chol	MC3	mRNA
MCH+ApoE3*	22 ± 0.2	55 ± 0.8	20 ± 0.9	3.00 ± 0.03	24.0 ± 0.6	76 ± 1	3.00 ± 0.02 10^{-2}
MCHPC+ApoE3*	18 ± 0.2	52 ± 0.8	27 ± 0.9	3.00 ± 0.02	20.0 ± 0.6	80 ± 1	4.00 ± 0.03 10^{-2}
MMC+ApoE3**	19 ± 0.2	57 ± 10	21 ± 3	3.0 ± 0.2	18.0 ± 1.3	82 ± 1	3.00 ± 0.04 10^{-2}
Average	20 ± 2	55 ± 2	23 ± 4	3.0 ± 0.1	21 ± 3	79 ± 3	3.3 ± 0.6 10^{-2}

*particle radius and shell thickness are smaller than prior to incubation.

**particle radius and shell thickness have same sizes as before incubation.

Protocol for LNP immobilization on QCM-D gold sensors and Binding isotherm for ApoE3 to LNP

Au-sensors (Q-sense) were cleaned in base piranha for 5 minutes at 75°C (5:1:1 H_2O:NH_3:H_2O_2), then rinsed with MilliQ water and dried under a stream of nitrogen, then the sensors were place in the O3-cleaner (UV ozone lamp) for 20 minutes and then immersed for at least 18 hours in 5mM biotin-PEG-thiol/PEG-thiol 5:95 mol% in EtOH. After incubation, the sensors were rinsed with EtOH and dried with nitrogen prior to mounting in the flow module of the QCM-D analyzer (Q-sense). Once the four sensors were mounted and equilibrated at 22°C in the flow modules, for each module: 1 ml of Streptavidin 25 µg/ml was flown at 100 µl/min, then rinsed with 1 ml PBS pH 7.4, 1 ml of AntiPEG-biotin 5 µg/ml was injected, then rinsed with 1 ml PBS, 1 ml of 50 µg/ml BSA was injected as blocking agent and a final 1ml of buffer was flown. The coverage of AntiPEG-AB is found to be $(2.07 \pm 0.14) \times 10^{12}\ \mathrm{mol} \cdot \mathrm{cm}^{-2}$ as will be reported in an upcoming publication (manuscript in preparation).

A peristaltic pump (IPC 4, Ismatec) was used for injection of all solutions.

LNP stock (mRNA concentration 0.1 mg/ml, all hydrogenous components) was diluted to 20 µg/ml (mRNA concentration) and 1ml was injected to each of the four flow modules. After injection, the solution was left for 1 hour prior to PBS rinsing (Figure SI4). Quick binding was observed accompanied by the formation of a soft layer (high increase in dissipation).

ApoE3 stock solution (0.5 mg/ml) was diluted to 0.5, 2.5, 5 and 12.5 µg/ml. After rinsing the LNP immobilized, 1ml of each ApoE dilution was injected to each sensor and left for about 10 minutes, then rinsed with PBS. Further adsorption occurred that did not induce a further increase in dissipation. Thus, no further softening of the adsorbed layer occurred upon ApoE

binding which justifies the use of the Sauerbrey equation for determination of ApoE adsorbed amount.[3]

Estimation of ApoE to LNP

From the wet mass adsorbed upon ApoE addition an upper limit for the number of molecules was determined by dividing the mass for the molecular weight (38 kDa). Considering an LNP of 60 nm in diameter to have a packing at the sensor surface to be random (60% coverage) or to have a hexagonal arrangement (90% coverage), we can determine the total LNP available surface on the sensor for ApoE binding and calculate the number of ApoE per LNP. From the number of ApoE per LNP we can determine the weight ratio estimating the LNP mass to be about 68 MDa for 60 nm diameter particle. For example 1:10 ApoE:lipid %w corresponds to about 180 ApoE per LNP.

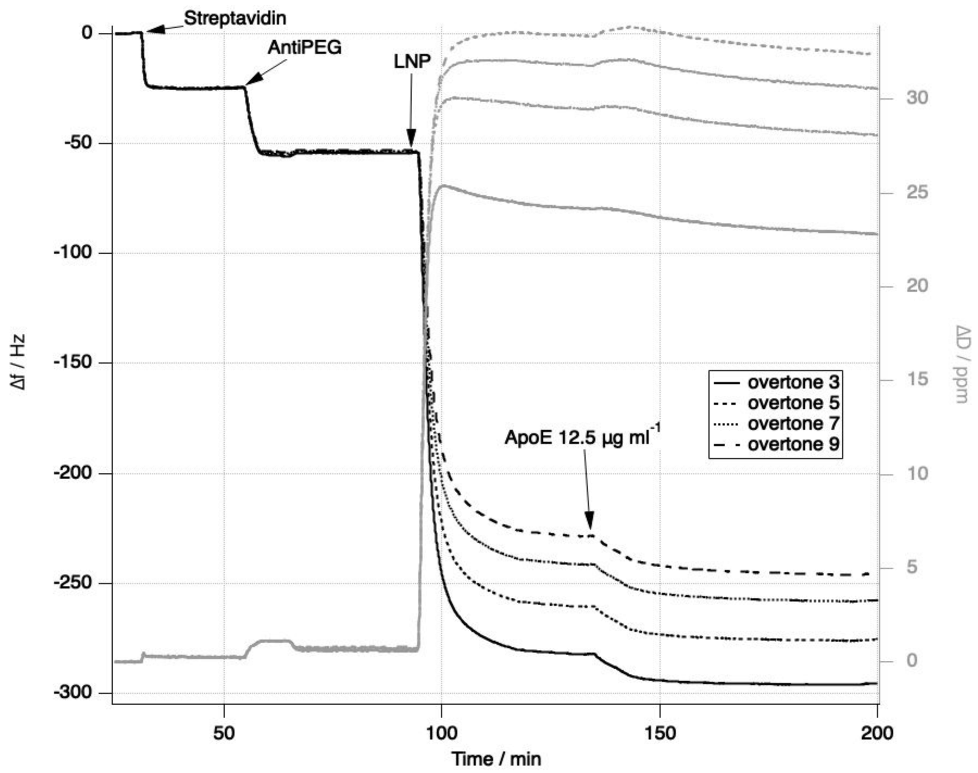

Figure SI4. Frequency shift (left y-axis, black) and dissipation (right y-axis, grey) recorded in the binding experiment of ApoE 12.5 µg/ml to immobilized LNP.

Additional SANS data: ApoE4 and HSA binding to LNP

In order to determine if the effect of ApoE3 binding to LNP is specific or general, we measured SANS data with ApoE4 and Human Serum Albumin (HSA).

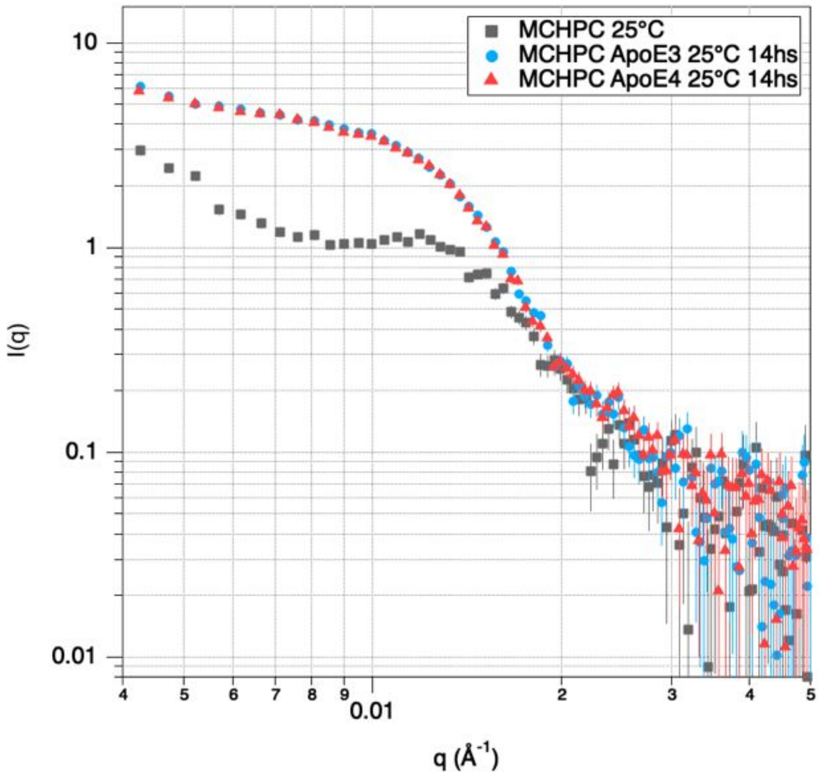

Figure SI5. SANS data collected for MCHPC (grey squares) incubated with ApoE3 (circles) and ApoE4 (triangles) at 25 °C upon 14 hours of incubation. All samples were diluted in 39% d-PBS. The effect on the LNP structure upon incubation with ApoE3 and ApoE4 is indistinguishable between isoforms.

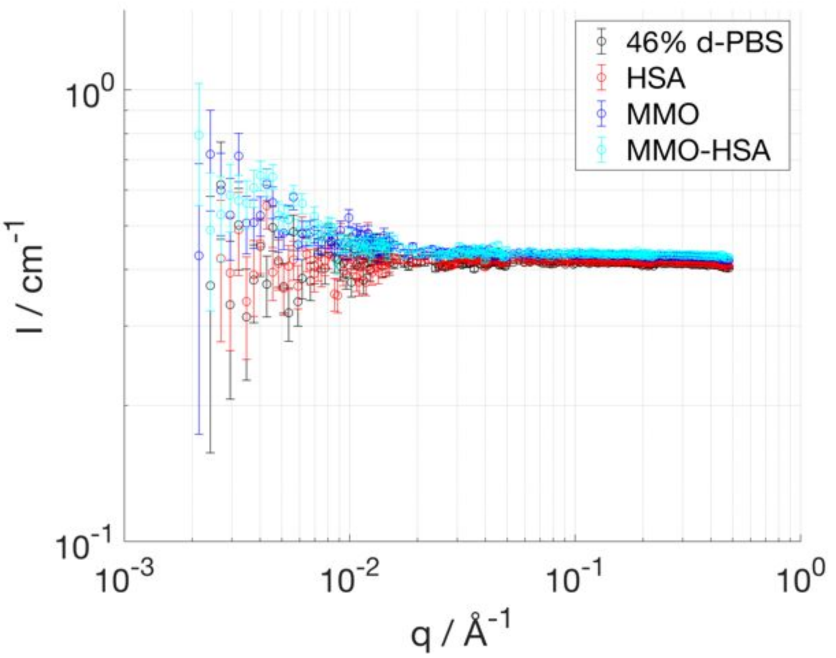

Figure SI6. SANS data collected in 46% d-PBS for: buffer (black), HSA (red), MMO (blue) and MMO incubated for 3 hours with HSA (light blue). All curves overlap within experimental errors, this suggests that HSA does not affect the structure and components distribution in the LNP to the same extent as apolipoprotein E.

To complement the SANS data shown for the ApoE incubated LNP, in Fig. SI7 SANS curves for ApoE3 in contrast matching conditions are reported.

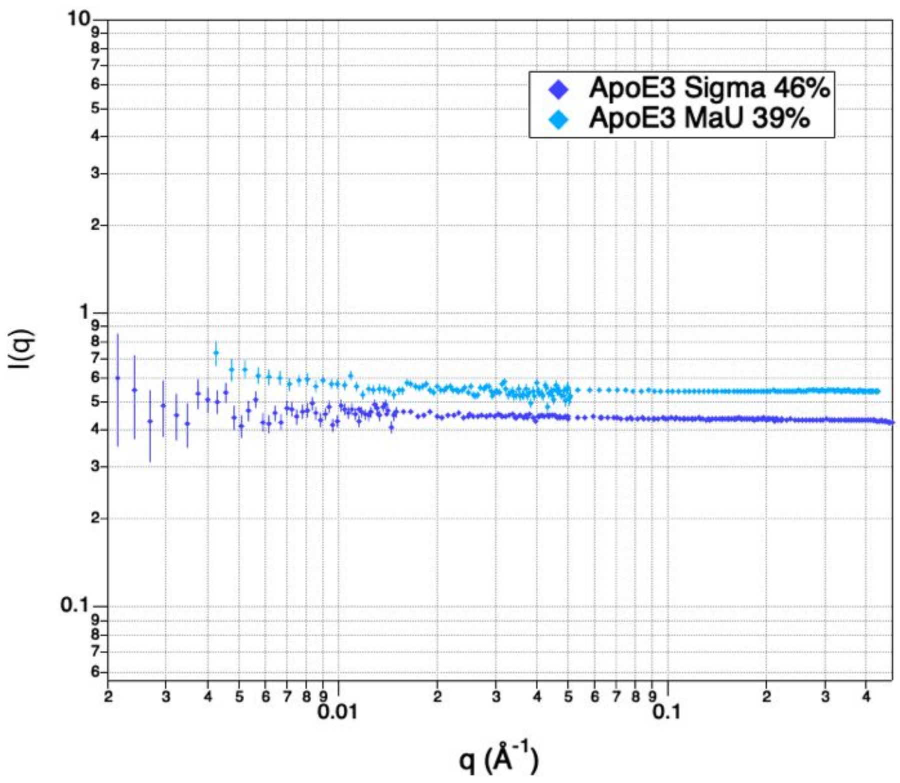

Figure SI7. SANS curves collected for ApoE3 0.3 mg/ml (blue symbol Sigma Aldrich, cyan symbol MaU) in solvents (39-46% d-PBS) close to the calculated contrast matching (43% d-PBS), data are not background subtracted.

SANS data for MCH/MCHPC in absence and presence of ApoE: Temperature study

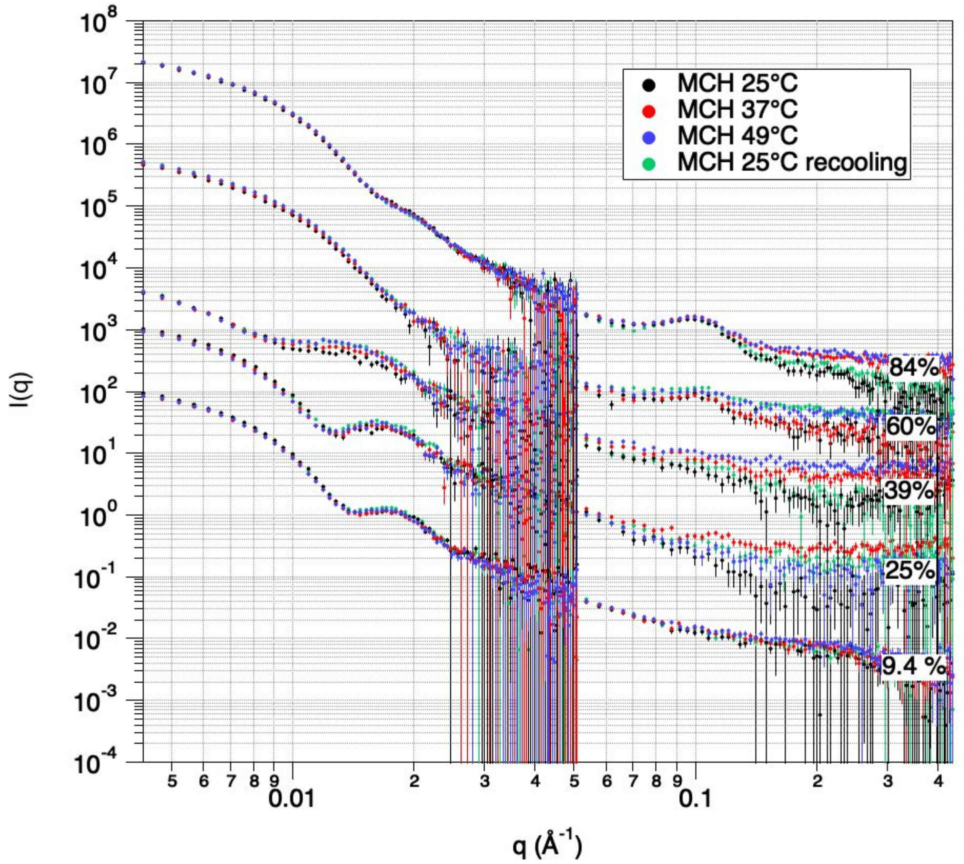

Figure SI8. SANS curve measured for MCH once sample equilibrated at 25, 37, 49 and then cooled at 25°C. Curves corresponding to the same solvent contrast are shifted for clarity. No visible structural effect due to heating is found.

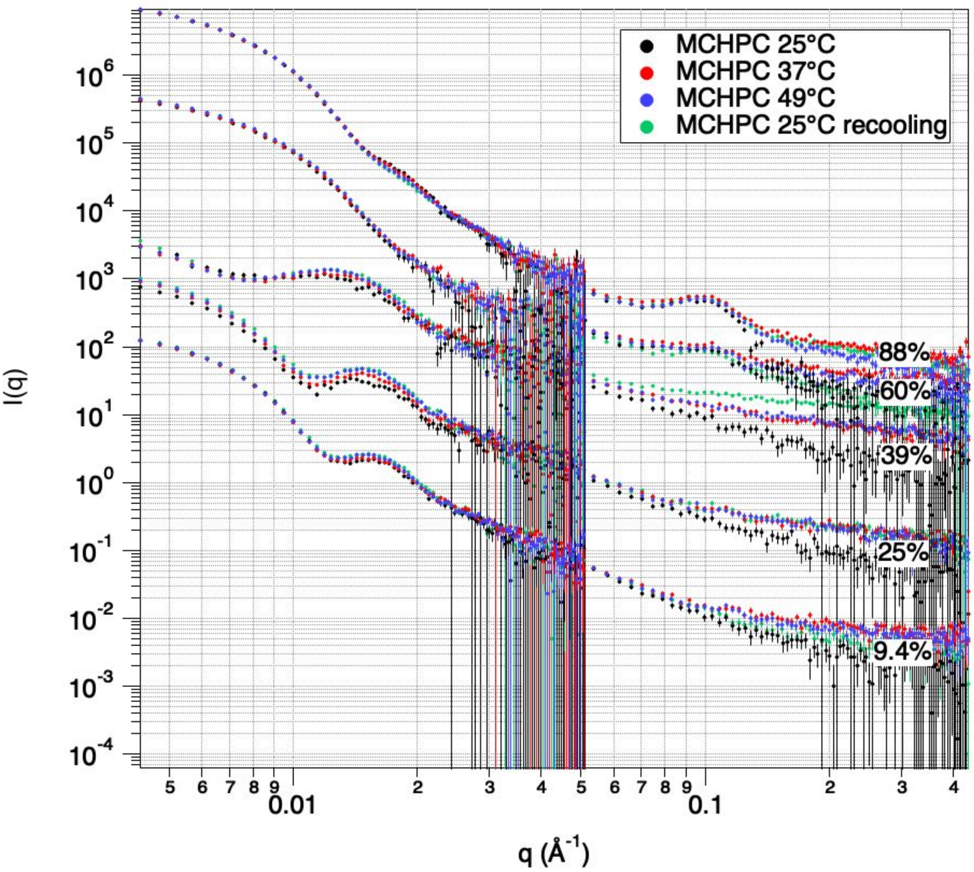

Figure SI9. SANS curve measured for MCHPC once sample equilibrated at 25, 37, 49 and then cooled at 25°C. Curves corresponding to the same solvent contrast are shifted for clarity. No visible structural effect due to heating is found.

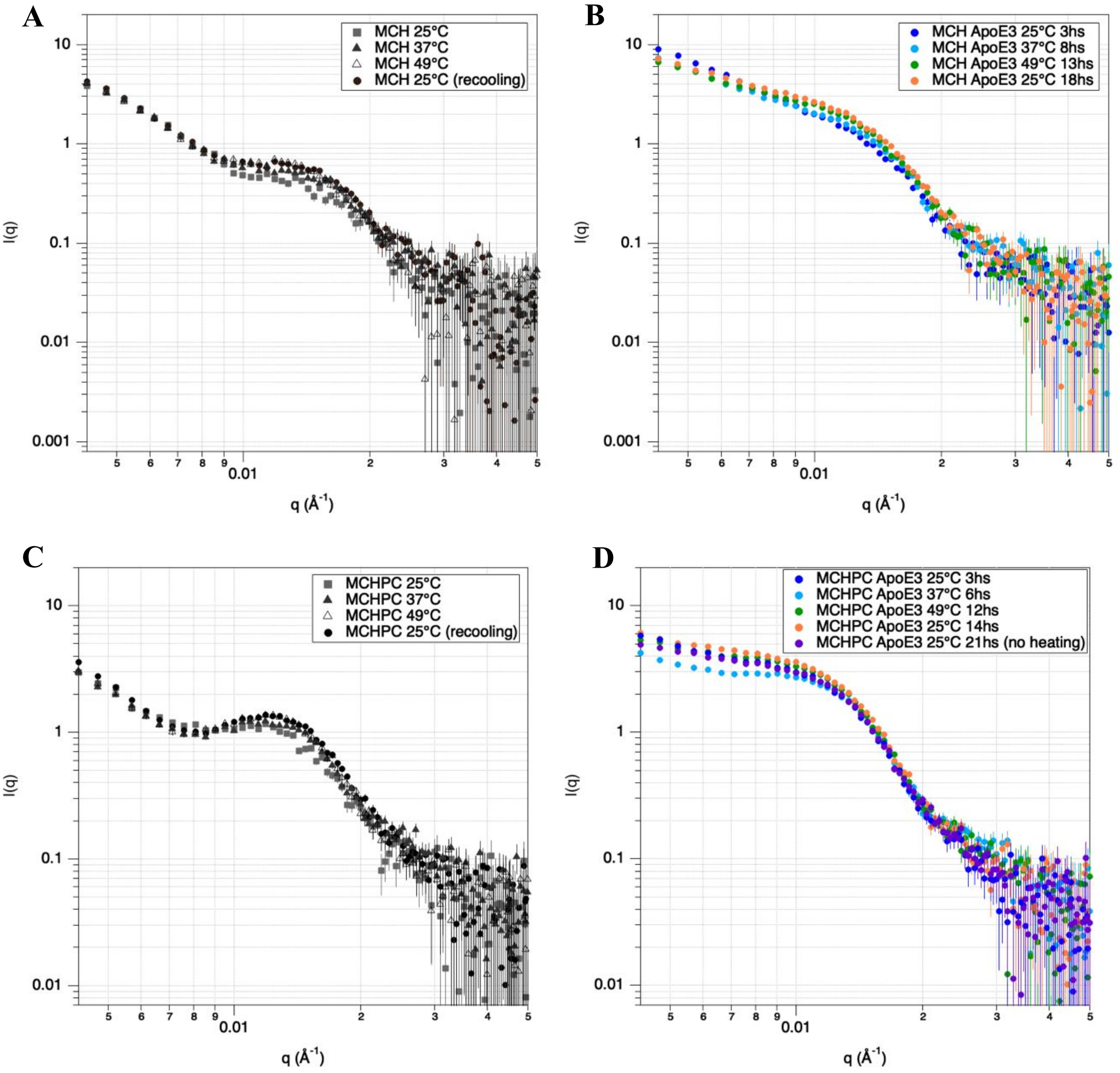

Figure SI10. SANS curves collected for MCH in absence (A) and presence (B) of ApoE3 as a function of T. SANS curves collected for MCHPC in absence (C) and presence (D) of ApoE3 as a function of T and compared to SANS curves collected for a sample left incubating at 25°C and measured after 21 hours. No visible difference is found between the sample incubated at 25°C and the one heated and then re-cooled to 25°C.

Subtraction of the MMC SANS data with and without ApoE in 46% dPBS

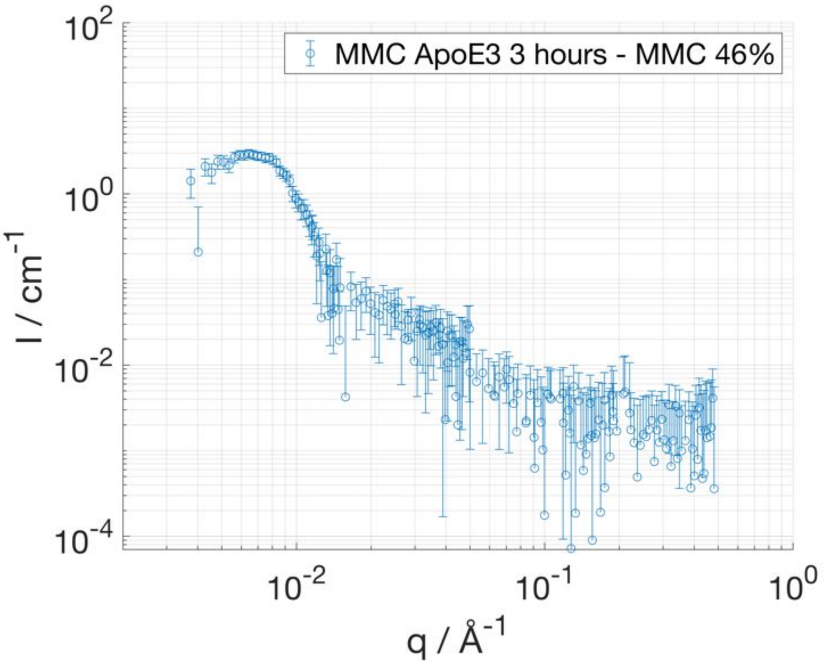

Figure SI11. Scattering intensity resulting from the subtraction of SANS data collected with MMC from SANS data collected with MMC incubated for 3 hours with ApoE (both in 46% dPBS). A peak around 0.006 Å^{-1} is clearly visible.

Analysis of the deuterium level of deuterated cholesterol (average 87% D)

Average mass of the sodium adduct of deuterated molecule (Fig. SI12A) by ESI-MS represented an increase of 39 mass units compared to protonated cholesterol (Fig. SI14). This equated to an average deuteration of 87% D (assumption of d_{45}=100%).

The ^{1}H and ^{2}H NMR spectra of cholesterol-d45 (Fig. SI12B) shows deuteration across all non-labile protons. By comparing the ^{1}H and ^{2}H NMR spectra and considering the relative ratios of the peaks, it is concluded that the alkene single proton at 5.3 ppm (C6) was deuterated more than the single proton at the C3 carbon next to the alcohol group at 3.5 ppm. The deuterium substitution (% D) of these positions and some others, shown in the structure below, were determined by integrating the ^{13}C signals in the ^{13}C {^{1}H,^{2}H} NMR spectra, which are isotopically shifted due to deuterium incorporation (Fig. SI13) using the method of Darwish *et al.*.[4] The rest of the positions were determined to be at 88% D based on the average deuterium level calculated for the whole molecule from the ESI-MS (87%D).

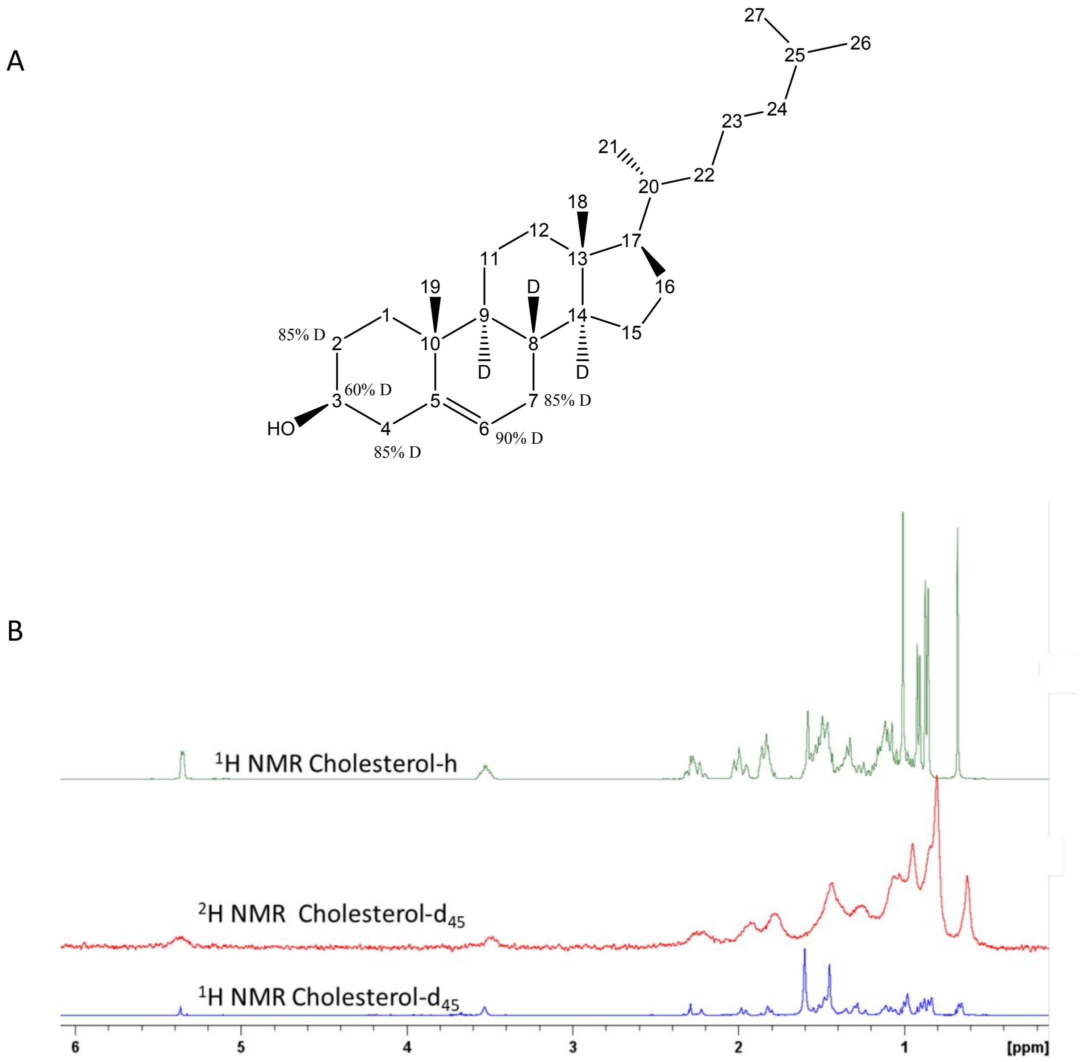

Figure SI12. Chemical structure of cholesterol-d$_{45}$ (A). ^{1}H NMR (400 MHz, $CDCl_3$) spectrum of Sigma-Aldrich protonated cholesterol standard (top, green); ^{2}H NMR (61.4 MHz, $CDCl_3$) spectrum of cholesterol-d$_{45}$ (red, middle), ^{1}H NMR (400 MHz, $CDCl_3$) spectrum of cholesterol-d$_{45}$ (bottom, blue) (B).The signal at 1.6 ppm in the ^{1}H NMR spectra corresponds to water.

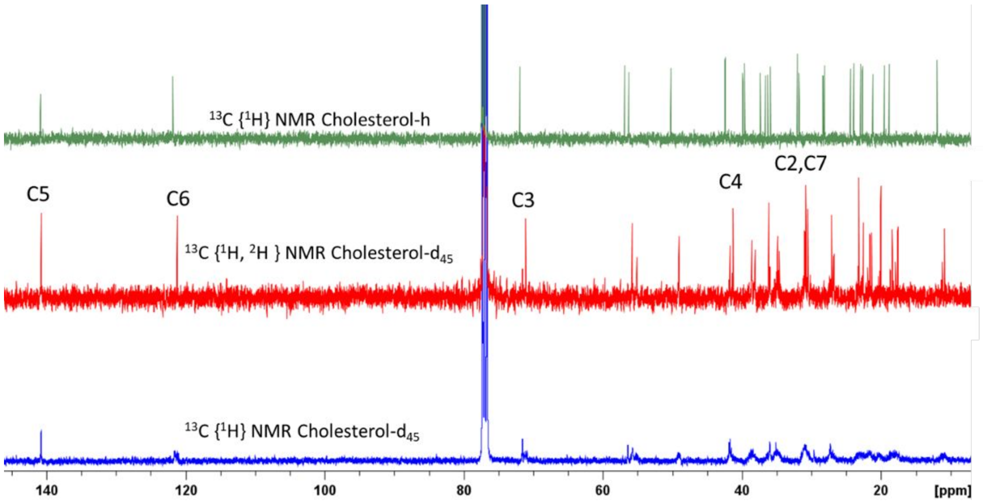

Figure SI13. ^{13}C NMR (100.6 MHz, $CDCl_3$) spectrum of Sigma-Aldrich protonated cholesterol standard (green, top); ^{13}C {^{1}H, ^{2}H decoupled} of cholesterol-d_{45} (red, middle) with some carbon peaks assigned according; ^{13}C of cholesterol-d_{45} (blue, bottom).

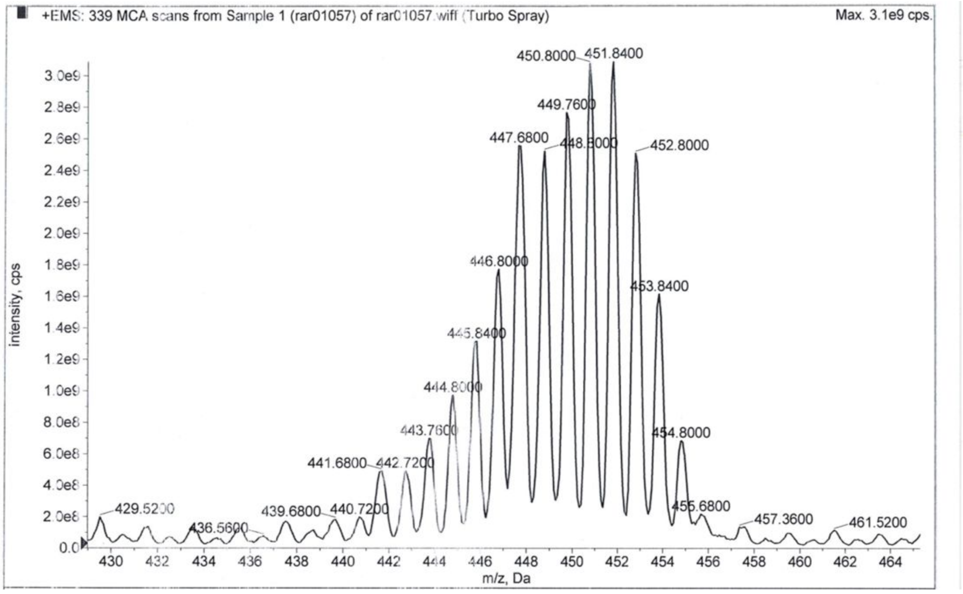

Figure SI14. ESI-MS (DP+50) of cholesterol-d_{45} (sodium adduct), where the major peak at 450.8 m/z indicates an increase of 41 mass units compared to protonated cholesterol. The average deuterium level is 87%D across isotopomers. ESI-MS isotopomers distribution: 2.1%, d_{45}; 5.8%, d_{44}; 9.2%, d_{43}; 11.6%, d_{42}; 11.9, d_{41}; 11%, d_{40}; 10%, d_{39}; 12.5%, d_{38}; 7.4%, d_{37}; 5.6%, d_{36}; 4.0%, d_{35}; 3.1%, d_{34}; 2.0%, d_{33}; 2.7%, d_{32}; 1.1%, d_{31}.

References

(1) Ehmann, H. M. A.; Spirk, S.; Doliška, A.; Mohan, T.; Gössler, W.; Ribitsch, V.; Sfiligoj-Smole, M.; Stana-Kleinschek, K. Generalized Indirect Fourier Transformation as a Valuable Tool for the Structural Characterization of Aqueous Nanocrystalline Cellulose Suspensions by Small Angle X-Ray Scattering. *Langmuir* **2013**, *29* (11), 3740–3748. https://doi.org/10.1021/la303122b.

(2) Glatter, O. Convolution Square Root of Band-Limited Symmetrical Functions and Its Application to Small-Angle Scattering Data. *J. Appl. Crystallogr.* **1981**, *14* (2), 101–108. https://doi.org/10.1107/S002188988100887X.

(3) Alassi, A.; Benammar, M.; Brett, D. Quartz Crystal Microbalance Electronic Interfacing Systems: A Review. *Sensors (Switzerland)* **2017**, *17* (12), 1–41. https://doi.org/10.3390/s17122799.

(4) Darwish, T. A.; Yepuri, N. R.; Holden, P. J.; James, M. Quantitative Analysis of Deuterium Using the Isotopic Effect on Quaternary (13)C NMR Chemical Shifts. *Anal. Chim. Acta* **2016**, *927*, 89–98. https://doi.org/10.1016/j.aca.2016.05.003.
